# ﻿Re-circumscription of the mimosoid genus *Entada* including new combinations for all species of the phylogenetically nested *Elephantorrhiza* (Leguminosae, Caesalpinioideae, mimosoid clade)

**DOI:** 10.3897/phytokeys.205.76790

**Published:** 2022-08-22

**Authors:** Shawn A. O’Donnell, Jens J. Ringelberg, Gwilym P. Lewis

**Affiliations:** 1 Department of Geography and Environmental Sciences, Northumbria University, Newcastle upon Tyne, NE1 8ST, UK Northumbria University Newcastle upon Tyne United Kingdom; 2 Department of Systematic and Evolutionary Botany, University of Zurich, 8008 Zurich, Switzerland University of Zurich Zurich Switzerland; 3 Accelerated Taxonomy Department, Royal Botanic Gardens, Kew, Richmond, TW9 3AE, UK Royal Botanic Gardens Richmond United Kingdom

**Keywords:** extrafloral nectaries, Fabaceae, generic delimitation, monophyly, nomenclature, taxonomy

## Abstract

Recent phylogenomic analyses of 997 nuclear genes support the long-held view that the genus *Entada* is congeneric with *Elephantorrhiza*. *Entada* is resolved as monophyletic only if the genus *Elephantorrhiza* is subsumed within it. The two genera were distinguished solely by relatively minor differences in the mode of dehiscence of the fruits (a craspedium separating into one-seeded endocarp segments in *Entada* versus a craspedium with the whole fruit valve breaking away from the persistent replum in *Elephantorrhiza*) and the craspedial fruit type itself provides a shared synapomorphy for the re-circumscribed *Entada*. Here, we provide a synopsis of *Entada*, including 11 new combinations in total, for the eight species, one subspecies and one variety previously placed in *Elephantorrhiza*, as well as a new combination for a subspecies of *Entadarheedei* Spreng. not previously dealt with when *Entadapursaetha* DC. was placed in synonymy. These new combinations are: *Entadaburkei* (Benth.) S.A. O’Donnell & G.P. Lewis, **comb. nov.**; *Entadaelephantina* (Burch.) S.A. O’Donnell & G.P. Lewis, **comb. nov.**; *Entadagoetzei* (Harms) S.A. O’Donnell & G.P. Lewis, **comb. nov.**; Entadagoetzeisubsp.lata (Brenan & Brummitt) S.A. O’Donnell & G.P. Lewis, **comb. nov.**; *Entadaobliqua* (Burtt Davy) S.A. O’Donnell & G.P. Lewis, **comb. nov.**; *Entadapraetermissa* (J.H. Ross) S.A. O’Donnell & G.P. Lewis, **comb. nov.**; *Entadarangei* (Harms) S.A. O’Donnell & G.P. Lewis, **comb. nov.**; Entadarheedeisubsp.sinohimalensis (Grierson & D.G. Long) S.A. O’Donnell & G.P. Lewis, **comb. nov.**; *Entadaschinziana* (Dinter) S.A. O’Donnell & G.P. Lewis, **comb. nov.**; *Entadawoodii* (E. Phillips) S.A. O’Donnell & G.P. Lewis, **comb. nov.**; and Entadawoodiivar.pubescens (E. Phillips) S.A. O’Donnell & G.P. Lewis, **comb. nov.** We provide a revised circumscription of the genus *Entada* which now comprises 40 species distributed pantropically, with the greatest diversity of species in tropical Africa. We present a complete taxonomic synopsis, including a map showing the global distribution of the genus and photographs showing variation amongst species in habit, foliage, flowers and fruits. A short discussion about extrafloral nectaries, mainly observed in the Madagascan species, is presented.

## ﻿Introduction

Traditional circumscriptions of the mimosoid genus *Entada* Adans. encompass around 30 species of large woody lianas to 75 m long, thin woody climbers, scandent shrubs, small trees and geoxylic suffrutices to < 0.5 m height, pantropical to subtropical in distribution and with a centre of species diversity in Africa south of the Sahara (Lungu 1995; Lewis et al. 2005). The species are as diverse in their ecologies as they are in growth forms, with many species occupying lowland rainforest, especially in riverine and littoral habitats, while others are adapted to savannah grassland, open woodland or seasonally dry tropical forest. To illustrate this range in habit and ecology, consider the large palaeotropical lianas *Entadarheedei* Spreng. and *E.phaseoloides* (L.) Merr., which produce pods up to two metres in length from flowers less than a centimetre long, involving impressive post-pollination mechanical reinforcement of fruit-bearing structures. These species and those with large fruits and seeds thought to be closely related, are often found in riparian forest and along the landward fringes of mangroves associated with hydrochory, i.e. they have riverine and oceanic seed dispersal. Mature pods of these and most other *Entada* species, split transversely and break up into one-seeded segments that break away from the persistent woody frame (the replum) – a fruit type referred to as a craspedium. In the large-fruited species inhabiting riparian forest, these one-seeded articles often wash into rivers which then carry the buoyant seed-bearing envelopes downstream and out to sea, where they can drift on ocean currents for at least a year and remain viable (Ridley 1930, p. 284), enabling dispersal across enormous distances. In addition to landing on shores in the tropics conducive to establishment, seeds of *E.gigas* (L.) Fawc. & Rendle, *E.rheedei* and *E.phaseoloides* frequently wash up on temperate coasts in northern Europe (Nelson 1978; Cadée and Piersma 1990; Alm 2003), southern South Africa (Muir 1933) and southeast Australia (Smith 1991), well outside their known ranges and bioclimatic niche limits. Presence of fossilised seeds of *Entadapalaeoscandens* (Awasthi & Prasad) Antal & Awasthi in late Oligocene facies from New Zealand (Conran et al. 2014) and similar in Oligocene and Miocene units from India and Nepal (Awasthi and Prasad 1990; Antal and Awasthi 1993), suggest that *Entada* seeds have been drifting on ocean currents for tens of millions of years. Contrast this with the species adapted to the fire-prone savannahs and seasonal edaphic grasslands on poorly drained Kalahari sands of south-central Africa, such as the geoxylic *E.arenaria* Schinz, *E.hockii* De Wild. and *E.dolichorrhachis* Brenan, the woody tissues of which are confined to subterranean stems from which sprout annual aerial shoots. This ‘underground tree’ life history strategy apparently represents an adaptation to the fire-prone and frequently burnt savannah environments in which they live and to the nutrient-poor soils with impeded drainage on which they occur (White 1976; Maurin et al. 2014; Pennington and Hughes 2014).

Species of *Entada* form keystone elements of coastal ecosystems under threat from climate change (Wong et al. 2014). Roots of *Entada* species form nodules that house ‘rhizobia’-bacteria (Sprent 2009), facilitating nitrogen-fixation and soil enrichment which, in turn, enables *Entada* plants to colonise impoverished soils and promote ecological succession (e.g. Bush et al. 1995). Many species also have promising medicinal potential. *Entada* seeds, roots, stems and leaves are rich in bioactive compounds, especially saponins, explaining their ethnopharmacological and domestic uses in many indigenous African and Southeast Asian cultures (Lungu 1995, pp. 69–72), as well as their broader pharmaceutical and economic potential. For example, Fabry et al. (1998) demonstrated the antibacterial activity of bark extract from *E.abyssinica* Steud. ex A. Rich. against 105 bacterial strains; Cioffi et al. (2006) showed the capacity of saponins extracted from the roots of *E.africana* Guill. & Perr. to inhibit the development of pre-cancerous kidney cell lines; and Zheng et al. (2012) demonstrated in type 2 diabetic rats the antidiabetic effects of saponins extracted from the seeds of *E.phaseoloides*.

## ﻿Generic delimitation

Delimitation of the genus *Entada* has remained relatively stable since Brenan (1986) transferred the five species of his short-lived subgenus of *Entada*, *Acanthentada* Brenan to the genus *Adenopodia* C. Presl. This revised placement of these species was based on palynological (Guinet 1969) and wider morphological evidence from pollen dispersal unit, armature, petiolar nectaries, ovary indumentum and stylar morphology (Lewis and Elias 1981), published in the 15 years after Brenan tentatively described subgenus Acanthentada (Brenan 1966). Although species of *Entada* and *Adenopodia* share similar craspedial pods that break up to leave a persistent replum, species of *Adenopodia* disperse their pollen as polyads (vs. monads in *Entada*), have prickles on stems and leaves (*Entada* are unarmed, save for *E.spinescens* Brenan which has spinescent stipules), display petiolar nectaries (absent in *Entada*, although see ‘Note on extrafloral nectaries’ below), have pubescent ovaries (glabrous in *Entada*) and styles that taper to a porate stigma (vs. tubular to cupuliform in *Entada*). Additionally, Brenan (1966, 1986) noted that the epicarp of *Adenopodia* pods remains attached to the endocarp, whereas these structures separate in the mature pods of *Entada*. In their review of the genera of tribe Mimoseae, Lewis and Elias (1981) used this suite of characters to argue for a more parsimonious placement of *Adenopodia* within the informal Piptadenia group of genera. This placement of *Adenopodia*, separate from *Entada* in the *Piptadenia*-containing Mimosa clade (*sensu*Koenen et al. 2020), is supported by recent large-scale phylogenomic analyses (Koenen et al. 2020; Ringelberg et al. 2022). In that same review of Mimoseae genera, Lewis and Elias (1981) highlighted the close affinity between *Entada* and *Elephantorrhiza* Benth., an essentially southern African genus of eight species of geoxylic suffrutices, shrubs or small trees and placed them in their informal Entada group. Both genera share craspedial fruits, leaflets in mostly opposite pairs, a perigynous stemonozone, pollen released as monads and a tubular to cup-shaped stigma. The primary distinguishing character is that the craspedia in species of *Elephantorrhiza* lack the transverse septa between seeds that are present in *Entada* and, hence, do not split into one-seeded segments along these septa upon ripening as in *Entada*. Rather, in *Elephantorrhiza*, the two valves separate from the replum, the epicarp usually peeling off the endocarp, with the valves otherwise remaining entire or breaking up irregularly. These two types of craspedia are also found within the large monophyletic genus *Mimosa* (Simon et al. 2011; Ringelberg et al. 2022), where species with craspedia in which the entire valve breaks away from the replum are phylogenetically deeply nested within the genus (Simon et al. 2010), just as found here in *Entada* / *Elephantorrhiza*, suggesting this switch is an evolutionarily easy one in mimosoid fruits and that this character is not useful for generic delimitation.

**Figure 1.**
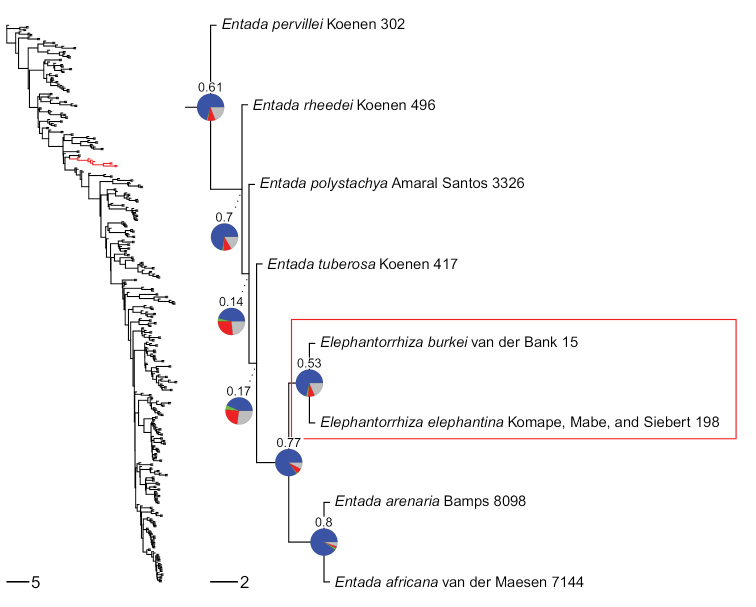
Non-monophyly of *Entada*, based on the ASTRAL (Zhang et al. 2018) single-copy genes phylogeny of Ringelberg et al. (2022). Red branches in the Caesalpinioideae subfamily-wide tree at left denote the phylogenetic position of the magnified *Entada* + *Elephantorrhiza* subtree at right. Red rectangular outline highlights the nested *Elephantorrhiza* species. Pie charts on nodes show the fraction of gene trees supporting each bipartition in blue, the fraction of gene trees supporting the most likely alternative configuration in green, the fraction of gene trees supporting additional conflicting configurations in red and the fraction of uninformative gene trees in grey, based on a total of 821 unique gene trees. Numbers above pie charts are Extended Quadripartition Internode Certainty scores (Zhou et al. 2020). Branch lengths are expressed in coalescent units and terminal branches were assigned an arbitrary uniform length for visual clarity. Tree: J Ringelberg.

Molecular phylogenetic analyses over the past twenty years have repeatedly supported a close relationship between *Entada* and *Elephantorrhiza* (e.g. Luckow et al. 2003; LPWG 2013, 2017; Koenen et al. 2020; Ringelberg et al. 2022), with generally greater resolution achieved as locus- and taxon-sampling and tree-building methods have improved. Luckow et al.’s (2003) strict-consensus tree of 134 mimosoid taxa, based upon two chloroplast regions, recovered *Elephantorrhizaelephantina* (Burch.) Skeels and *Entadaabyssinica* as sister species, the pair, in turn, sister to *Entadarheedei*, suggesting that *Elephantorrhiza* might be nested within *Entada*. The LPWG (2017) family-wide *matK* phylogeny included eight species of *Entada* and three species of *Elephantorrhiza* and also showed the latter to be phylogenetically nested within *Entada*. Ringelberg et al.’s (2022) phylogenomic analyses of subfamily Caesalpinioideae (*sensu*LPWG 2017) used 997 nuclear genes and included eight species from across the two genera, again showing robust support for *Elephantorrhiza* being nested within *Entada* (Fig. 1). The combined morphological and molecular evidence thus overwhelmingly supports sinking *Elephantorrhiza* into *Entada*.

We present a synopsis of the here re-circumscribed genus *Entada*, including a synthesis of species descriptions from existing literature, and propose new combinations in *Entada* for all eight species of *Elephantorrhiza*, based upon the strong molecular evidence discussed above. This formal transfer of species resolves the non-monophyly of traditional circumscriptions of *Entada*.

## ﻿Taxonomy

### ﻿ Entada

Taxon classificationPlantaeFabalesFabaceae

Adans., Fam. Pl. 2: 318. 1763, emended S.A. O`Donnell & G.P. Lewis.

8870DBC6-8402-5AEF-B3E7-01822736F17E

Gigalobium P. Browne, Civ. Nat. Hist. Jamaica: 362. 1756.
Perima Raf., Sylva Tellur.: 118. 1838.
Strepsilobus Raf., Sylva Tellur.: 117. 1838.
Elephantorrhiza Benth., J. Bot. (Hooker) 4: 344. 1841. Synon. nov.
Pusaetha L. ex Kuntze, Revis. Gen. Pl. 1: 204. 1891.
Entadopsis Britton, N. Amer. Fl. 23: 191. 1928.

#### Type species.

*Entadarheedei* Spreng.

#### Description.

**Figure 2.**
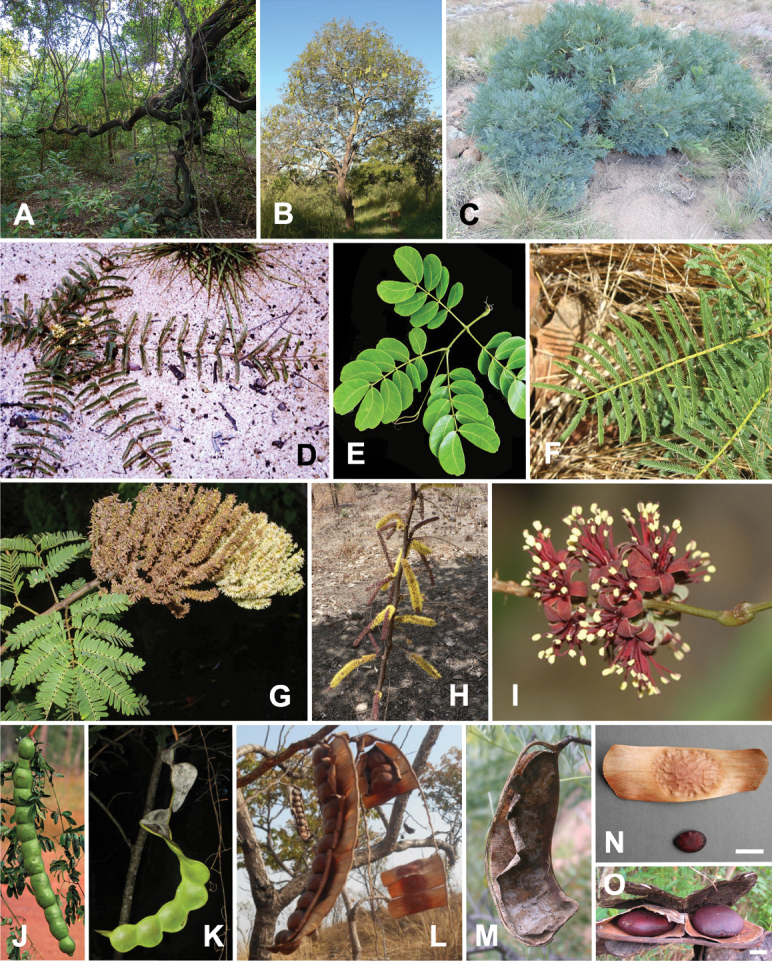
Genus-wide variation in morphological characters. **A–D** habit **A** large woody liana, *E.rheedei* (photo: B Wursten, Hyde et al. (2021)) **B** small tree, *E.abyssinica* (photo: G Baumann, Dressler et al. (2014a)) **C** shrub, *E.burkei* (photo: M Schmidt, Dressler et al. (2014a, b)) **D** geoxylic suffrutex, *E.dolichorrhachis* (photo: M Bingham, Bingham et al. (2021)) **E, F** leaves **E** bipinnate leaf with few pinnae, few large leaflets and ending in a bifurcating tendril, *E.rheedei* (photo: AP Balan, Balan and Predeep (2021)) **F** bipinnate leaf with many pinnae, many small leaflets and no tendril, *E.rangei* (photo: A Dreyer, Dressler et al. (2014a)) **G–I** inflorescences **G** terminal panicle of up-turned spikes, *E.polyphylla* (photo: R Vásquez Martínez, CC BY-NC-SA 3.0, MBG (2021)) **H** axillary fascicles of spiciform racemes, *E.goetzei* (photo: G Baumann, Dressler et al. (2014a)) **I** short spiciform raceme of dark red flowers, *E.wahlbergii* (photo: R Mangelsdorff, Dressler et al. (2014a)) **J–M** fruits **J** immature, weakly falcate, segmented craspedium up to 2 m long, *E.rheedei* (photo: photographer unknown, Centre for Australian National Biodiversity Research (CANBR), 2000) **K** immature, segmented, laxly spiralled craspedium up to 120 cm long, *E.gigas* (photo: R Aguilar CC BY-NC-SA 2.0, Aguilar (2021)) **L** ripe segmented craspedia breaking up into one-seeded segments with exfoliating epicarp, *E.africana* (photo: B Eichhorn, Dressler et al. (2014a)) **M** ripe unsegmented craspedium, the entire valve breaking away from the persistent replum, *E.burkei* (photo: M Kriek CC BY-SA 4.0, Ueda (2021) observation 85675968) **N, O** seeds **N** one-seeded endocarp segment and small ovoid, flattened seed with elliptic pleurogram, *E.africana* (photo: B Eichhorn, Dressler et al. (2014a)) **O** ripe one-seeded fruit segments with large circular, laterally compressed seeds lacking a pleurogram, *E.gigas* (photo: J Stevens, Dressler et al. (2014a)). Scale bars: 1 cm (**N, O**).

Lianas, scandent shrubs, small trees or geoxylic suffrutices, unarmed or with spinescent stipules in *E.spinescens*. **Leaves**: bipinnate; primary and secondary axes either eglandular or, in some Madagascan species, with extrafloral nectaries (see Note below) and at least in *E.phaseoloides*, with unusual ‘pit’ nectaries on stems at nodes adjacent to petiole; rachis in lianescent taxa terminating in a bifurcating tendril (modified terminal pinnae pair); pinnae 1–many pairs per leaf; leaflets 1–many pairs per pinna; lamina often asymmetric and apically mucronate or emarginate. **Inflorescence**: spiciform racemes or spikes, axillary to supra-axillary, solitary or clustered, sometimes into terminal panicles. **Flowers**: 5-merous, sessile to shortly pedicellate, staminate or bisexual, cream-coloured, yellow, green, red or purple; calyx gamosepalous, campanulate, the fused sepals distinctly toothed or not; petals 5, free to basally connate, adnate basally with the stamens and a perigynous disc forming a stemonozone; stamens 10, fertile, free or basally united, anthers usually with a caducous spheroidal apical gland, sessile to stipitate; pollen tricolporate, columellate, dispersed as monads; style tapering to a tubular to rarely cupuliform stigma, ovary glabrous and multi-ovulate. **Fruit**: a craspedium, torulose or not, compressed to flattened, straight to curved to rarely spirally twisted, sometimes gigantic (up to 2 m long in taxa with sea-drifted seeds); epicarp woody to thinly coriaceous; endocarp woody to parchment-like; splitting along transverse septa into one-seeded segments upon ripening or valvately dehiscent, the entire valve breaking away from the replum and the epicarp also separating from the endocarp. **Seeds**: globular to elliptic, usually laterally compressed, longest axis up to 6 cm in large-fruited taxa, dark brown, smooth, with or without areole, pleurogram (when present) usually open. Fig. 2.

**Figure 3.**
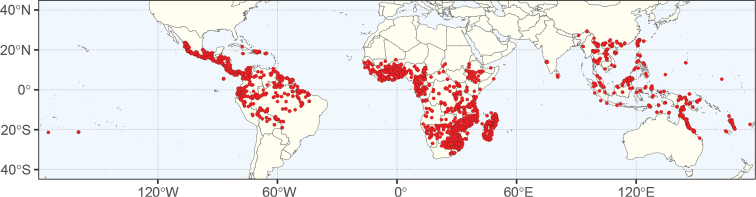
Global distribution of 4415 digitised, quality-controlled herbarium records of *Entada* (including those for ex-*Elephantorrhiza* species) in GBIF (www.GBIF.org), DryFlor (www.dryflor.info) and SEINet (swbiodiversity.org/seinet) from Ringelberg et al. (in prep.). Map created using R packages ggplot2 (Wickham 2016), sf (Pebesma 2018) and rnaturalearth (South 2017). Map: J Ringelberg.

As delimited here, a genus of 40 species (traditionally ± 30 species), widespread, primarily tropical, but reaching subtropical latitudes in southern Africa and eastern Asia (Fig. 3); 29 species in Africa (including Madagascar), nine species in Asia, four species in the Americas; two species (*E.rheedei* and *E.gigas*) occur in two of these regions. Frequently found in riparian and littoral vegetation, though also in savannah, open woodland, thickets or dense humid to more open and dry forest, often on sandy substrates.

##### ﻿Note on extrafloral nectaries

**Figure 4.**
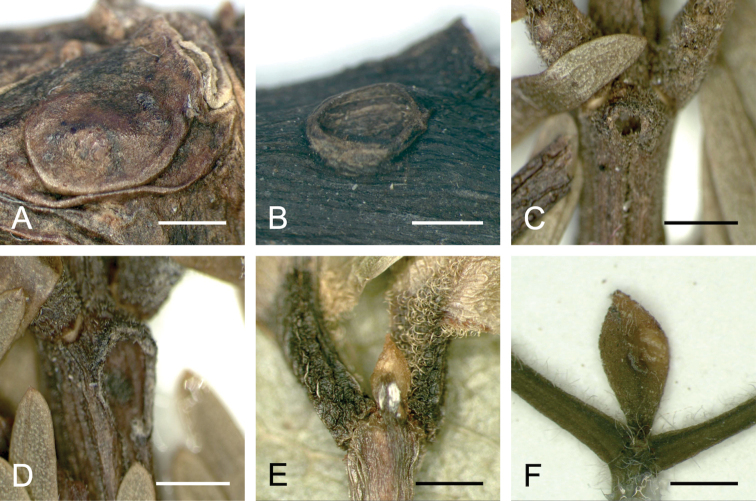
Putative extrafloral nectaries on herbarium specimens of five of the six native Madagascan species of *Entada***A***E.leptostachya, Du Puy et al. M235***B***E.louvelii, Réserves Naturelles RN1447***C***E.pervillei, Service Forestier 10525_SF***D***E.pervillei, Service Forestier 11481_SF***E***E.rheedei, Gautier LG3153***F***E.tuberosa, Jongkind et al. 3264*. Scale bars: 2 mm (**A–F**). Photos: S O’Donnell.

**Figure 5.**
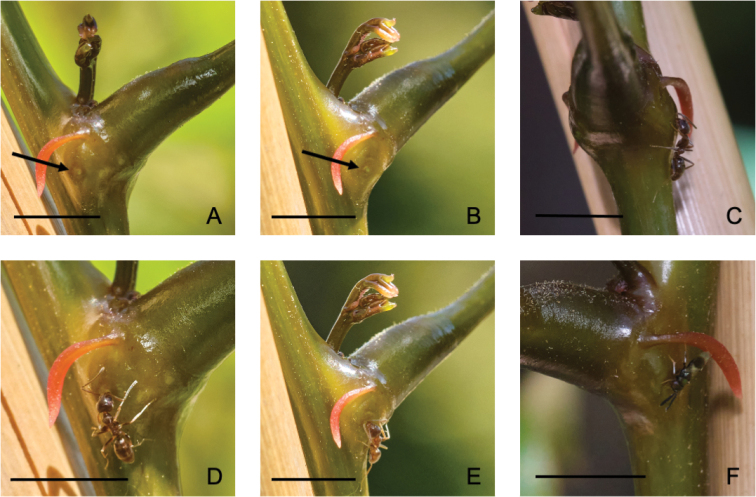
Extrafloral nectaries at nodes on stems of cultivated *Entadaphaseoloides* grown in California from seed. **A, B** Pit nectary at node on stem adjacent to petiole, indicated by arrow **C–E** Argentine ants (*Linepithemahumile*) visiting nectaries **F** dipteran nectary visitor. Scale bars: 5 mm (**A–F**). Photos: S O’Donnell.

While much of the literature on *Entada* (e.g. Brenan 1966; Lewis and Elias 1981; Nielsen 1981, 1992; Villiers 2002; Wu and Nielsen 2010; Braga et al. 2016) noted the absence of petiolar and leaf rachis nectaries that are otherwise common across the mimosoid clade, examination of herbarium specimens from Madagascar uncovered several species that do appear to possess putative extrafloral nectaries. Six species of *Entada* are native to Madagascar (Villiers 2002): *E.chrysostachys* (Benth.) Drake; *E.leptostachya* Harms; *E.louvelii* (R. Vig.) Brenan; *E.pervillei* (Vatke) R. Vig.; *E.rheedei* Spreng.; and *E.tuberosa* R. Vig. Of these, *E.louvelii*, *E.pervillei* and *E.tuberosa* are endemic to the Island. Villiers (2002) noted that *E.tuberosa* “is easily recognisable by the white, glandular mucro at the tip of the leaf rachis and the axes of the pinnae (generally present)” (Villiers 2002, p. 169). Close examination of specimens at K reveals structures that are here interpreted as extrafloral nectaries on five of the six Madagascan *Entada* species (*E.chrysostachys* is the only species on which these structures were not observed) (Fig. 4). These nectaries are visible as annular structures on shoots immediately beneath the base of stipules, in similar positions to those documented for *E.phaseoloides* (Blüthgen and Reifenrath 2003; Marazzi et al. 2019) (Fig. 5), in all five potentially extrafloral nectary-bearing species. In addition, multiple vouchers of *E.pervillei* contain material with small basin-like structures at the distal end of adaxially grooved rachises that are also interpreted here as nectaries (Fig. 4C, D). Finally, structures comparable to those described by Villiers (2002) for *E.tuberosa* and confirmed on several K vouchers ascribed to this species (e.g. Fig. 4F), were also observed on specimens identified as *E.rheedei* (Fig. 4E). Examination of living material, chemical analyses of any exudates that might issue from all the above-mentioned structures and observations of animal visitation are needed to verify the interpretation offered here that these structures are indeed nectaries. The presence of these structures begs the question about how widespread they might be across the genus. A detailed study of extrafloral nectaries across the full geographical range of *Entada* should be carried out, this using a high-powered microscope and backed up by fieldwork.

We present no infrageneric classification at this point, pending a more densely sampled species-level molecular phylogeny and more detailed taxonomic revision which are foci of proposed future work. Instead, species are here simply alphabetically ordered. Species descriptions, species delimitation and synonymy are based on regional floristic treatments in Brenan (1959, 1966, 1970), Ross (1974, 1975a, 1975b), Nielsen (1992), Barneby (1996), Cowan (1998), Villiers (2002), Tateishi et al. (2008), Wakita et al. (2008), Ohashi et al. (2010), Wu and Nielsen (2010), Grobler (2012) and Braga et al. (2016), as well as Lungu’s (1995) global synopsis and, for the ex-*Elephantorrhiza* species, rely almost entirely upon Ross (1974, 1975a) with occasional additions from Brenan (1970) and Grobler (2012). Where opinions in literature differ, we defer to Brenan (1959, 1966, 1970), Ross (1974, 1975a), Nielsen (1992) and Villiers (2002) with any exceptions noted in the corresponding species descriptions. In addition to basionyms, we include synonyms only when these are names published in the genus *Entada*. For example, under *Entadaabyssinica*, we do not present the synonyms published in the genera *Pusaetha*, *Gigalobium* or *Entadopsis*. We include type details for all accepted species, but not for synonyms.

### ﻿ Entada abyssinica

Taxon classificationPlantaeFabalesFabaceae

Steud. ex A. Rich, Tent. Fl. Abyss. 1: 234. 1847.

4F6C3C51-77C2-56A7-AB88-39B11BF2FC6D

= Entada abyssinica var. microphylla Oliv., Fl. Trop. Afr. 2: 228. 1871. Synon. nov.
= Entada abyssinica var. intermedia Fiori, L’Agricoltura Colon. 5: 170. 1911. Placed as a synonym of E.abyssinica by Thulin (1983) in Leguminosae of Ethiopia: 36. 1983.

#### Types.

ETHIOPIA. Tigray region, mountains of Shire Dschogardi, *Schimper 520* (isosyntypes: BR [BR0000008378606], H [H1034939], HAL [HAL0120946], K [000232163, 000232164], LG [LG0000090027161], M [0108317], MO [MO-954247], MPU [MPU016174], P [P00418276, P00418277 & P00418278], S [S13-12046], TUB [TUB000996 & TUB000997]); ETHIOPIA. Abyssinie, *Quartin Dillon s.n*. (syntype: MPU [MPU016240 & MPU016246]).

#### Description.

**Figure 6.**
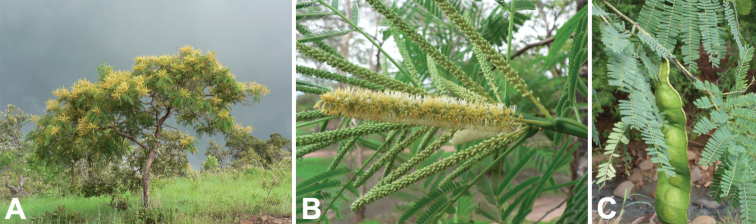
*Entadaabyssinica* habit, vegetative and reproductive structures. **A** small tree, Malawi (photo: G Baumann, Dressler et al. (2014a)) **B** spiciform racemes both pre- and post-anthesis, Malawi (photo: G Baumann, Dressler et al. (2014a)) **C** immature fruit and leaves, Burkina Faso (photo: A Thiombiano, Dressler et al. (2014a)).

Tree 2.7–10(–15) m tall, crown spreading (Figs 2B, 6A). **Leaves**: rachis 16.3–21.7 cm long, tendrils absent; pinnae 12–20 pairs per leaf, each pinna 4.8–7.8 cm long, with 20–55 pairs of leaflets; leaflets 4–12 × 1–3 mm, linear-oblong, apex rounded to obtuse and mucronate, base rounded to sub-truncate, mid-rib oblique, closer to the distal margin, lamina appressed-pubescent above and below though sometimes glabrescent above (Fig. 6C). **Inflorescence**: a 7–16 cm long spiciform raceme, either solitary or in groups of up to 4 inserted in a supra-axillary position, inflorescence peduncle and rachis pubescent (Fig. 6B). **Flowers**: creamy white turning yellowish, sweetly scented, pedicels 0.5–1 mm long; calyx 0.75–1 mm long, shallowly toothed, glabrous; petals 1.5–3 × 1 mm; stamen filaments 3.5–6 mm long (Fig. 6B). **Fruit**: a laterally compressed, torulose, almost straight craspedium, 15–39 × 3.8–9 cm, with transverse septa between seeds dividing the fruit into one-seeded segments which, upon ripening, fall from the persistent replum; segments moderately umbonate over seeds (Fig. 6C). **Seeds**: 1–1.3 × 0.8–1 cm, pleurogram elliptic, C-shaped or closed.

#### Distribution.

Tropical and southern subtropical Africa (excluding Madagascar).

#### Habitat and ecology.

Wooded grassland (Chipya), fringes of woodland (Miombo, characterised by *Brachystegia* Benth.), riparian vegetation and – in Sierra Leone – on laterite plateaux; 430–2290 m alt.

### ﻿ Entada africana

Taxon classificationPlantaeFabalesFabaceae

Guill. & Perr., Fl. Seneg. Tent.: 233. 1832.

12EC600F-0170-52B2-9DF9-DC209D87B07E

= Entada ubanguiensis De Wild., Pl. Bequaert. 3: 88. 1925.
= Entada sudanica Schweinf., Reliq. Kotschy.: 8. 1968.

#### Types

**(fide Brenan 1959: 12).** SENEGAL. Tiélimane, Cayor, *Leprieur* (syntype: G; photo: K); GAMBIA. Albreda, *G.S. Perrottet 290* (isosyntypes: BM [BM000842201], G; photo: K).

#### Description.

**Figure 7.**
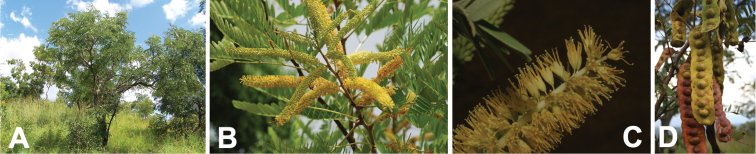
*Entadaafricana* habit, vegetative and reproductive structures. **A** small tree, Burkina Faso (photo: A Gockele, Dressler et al. (2014a)) **B** spiciform racemes both pre- and post-anthesis, Côte d’Ivoire (photo: S Porembski, Dressler et al. (2014a)) **C** open, pedicellate flowers, Mali (photo: P Birnbaum, Dressler et al. (2014a)) **D** mature fruits at varying ripeness, Burkina Faso (photo: Marco Schmidt, Dressler et al. (2014a)).

Shrub to small tree, 1.2–10 m tall, bark very rough (Fig. 7A). **Leaves**: variable, rachis 5.3–30 cm long, tendrils absent; pinnae 2–10 pairs per leaf, each pinna 7.1–17 cm long, with 10–24 pairs of leaflets; leaflets 1–3.1 × 0.32–0.85 cm, linear-oblong to elliptic- or obovate-oblong, apex rounded, base obtuse to oblique, mid-rib sub-central above base, lamina glabrous to slightly puberulous. **Inflorescence**: a 6.5–15 cm long, spiciform raceme, either solitary or in groups of up to 4 inserted in a supra-axillary position, peduncle and rachis usually glabrous, rarely pubescent (Fig. 7B). **Flowers**: yellow to white, sweetly scented, pedicels 1(–1.5) mm long; calyx 0.75–1.25 mm, shallowly toothed, glabrous; petals 1.5–4 × 0.6–1 mm (Fig. 7C). **Fruit**: a torulose, laterally compressed, almost straight craspedium, 38 × 5–7.3 cm; with transverse septa between seeds dividing the fruit into one-seeded segments which, upon ripening, fall from the persistent replum; segments distinctly umbonate over seeds (Figs 2L, 7D). **Seeds**: ovoid, 1.2 × 0.9–1 cm (Fig. 2N).

#### Distribution.

Throughout tropical sub-Saharan Africa, north of the equator.

#### Habitat and ecology.

Savannah grasslands and woodland, often in association with *Terminalia* L., *Combretum* Loefl., *Philenopteralaxiflora* (Guill. & Perr.) Roberty and *Pterocarpuslucens* Lepr. ex Guill. & Perr. (Lungu 1995, p. 35).

### ﻿ Entada arenaria

Taxon classificationPlantaeFabalesFabaceae

Schinz, Mém. Herb. Boissier 8: 118. 1900.

50461AFE-F761-5515-B439-D818B1CA837D

#### Type.

NAMIBIA. Hereroland, Grootfontein District, Omuramba-Omatako River, *Schinz 277* (holotype: Z).

#### Description.

**Figure 8.**
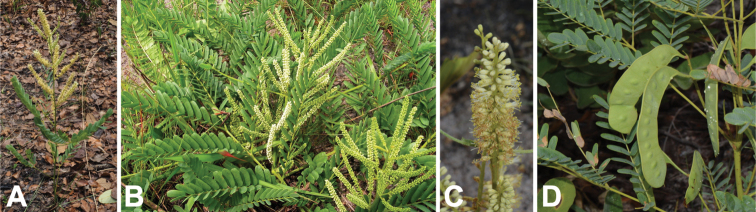
*Entadaarenaria* habit, vegetative and reproductive structures. **A** geoxylic suffrutex with erect annual stem, Angola (photo: D Goyder *CC BY-NC 4.0*, Ueda (2021) observation 35199077) **B** leaves and spiciform racemes pre-anthesis, Democratic Republic of Congo (photo: J Stevens, Dressler et al. (2014a)) **C** open, pedicellate flowers, Democratic Republic of Congo (photo: W McCleland, all rights reserved, iNaturalist (2021) observation 95512918) **D** leaves and immature fruits, Zambia (photo: W McCleland, Dressler et al. (2014a)).

Geoxylic suffrutex with erect annual 5–120 cm stems, young stems densely pubescent (Fig. 8A). **Leaves**: petiole 6–12 cm long, grooved above, puberulous; rachis 4–17 cm long, grooved above; pinnae 2–4 pairs per leaf, 7.5–14 cm long, with 6–13 pairs of leaflets; leaflets (1.2–)2–3.5(–4) × 0.7–2 cm, narrowly oblong to obovate-oblong, apex rounded to emarginate, base asymmetric, rounded to cordate on proximal margin, cuneate to cuneate-rounded on distal margin, lamina pubescent below at least on mid-rib and often throughout (Fig. 8A, B, D). **Inflorescence**: an axillary spiciform raceme 4–12 cm long, 1–3 per axil, rachis usually glabrous (Fig. 8A–C). **Flowers**: pale cream, pedicels 1–2 mm long; calyx campanulate, 1–2 mm long, shallowly toothed; petals 3–4 mm long; stamen filaments 5–6 mm long (Fig. 8C). **Fruit**: a torulose, laterally compressed, straight to distinctly falcate craspedium, 7.5–22 × 1.5–6 cm, with transverse septa between seeds dividing the fruit into one-seeded segments which, upon ripening, fall from the persistent replum (Fig. 8D). **Seeds**: 12.5 × 9 mm or smaller (see subsp. microcarpa below), dark brown, smooth.

### ﻿ subsp. arenaria

Taxon classificationPlantaeFabalesFabaceae

C2C4C906-8731-5F01-BD87-B37F27F7BA1E

= Entada nana Harms, Kunene–Sambesi Exped.: 244. 1903.

#### Description.

Stems 30–120 cm high. Fruit strongly falcate, 17–22 × 5–6 cm. Seeds 12.5 × 9 cm.

#### Distribution.

Namibia, Botswana, Zimbabwe, Zambia, Angola.

#### Habitat and ecology.

Woodland on Kalahari sand; ca. 900 m alt.

### ﻿ subsp. microcarpa

Taxon classificationPlantaeFabalesFabaceae

(Brenan) J.H. Ross, Bothalia 11: 126. 1973.

6309B381-35CE-5E31-A31D-7B6D28F7BB03

#### Type.

ZAMBIA. Mwinilunga District, Dobeka Bridge, *E. Milne-Redhead 4496* (holotype: K; isotype BR [BR0000006252199]).

#### Basionym.

EntadananaHarmssubsp.microcarpa Brenan, Kew Bull. 20(3): 373. 1966.

#### Description.

Stems 5–25 cm high. Fruit nearly straight, 7.5–12 × 1.5–2.8 cm. Brenan (1970, p. 20) noted that he did not see mature seeds, but presumed them to be smaller than in subsp. arenaria.

#### Distribution.

Zambia, Democratic Republic of Congo.

#### Habitat and ecology.

Grassland and woodland on Kalahari sand; ca. 1200 m alt.

### ﻿ Entada bacillaris

Taxon classificationPlantaeFabalesFabaceae

F. White, Bol. Soc. Brot., sér. 2, 33: 5. 1959.

59DD1AAB-F8F6-5C63-9E03-DF8C00E92C79

#### Type.

ZAMBIA. Abercorn District, Kambole escarpment, *H.M. Richards 9986* (holotype: K [K000232144, K000232145 & K000232146]; isotypes: BR [BR0000006251895 & BR0000006252229]).

#### Description.

Shrub 1.2–1.8 m tall, little-branched, young stems with golden to grey indumentum. **Leaves**: rachis 17–30 cm long, pubescent, tendrils absent; pinnae 3–4(–10) pairs per leaf, 10–17.5 cm long, with 8–13(–24) pairs of leaflets; leaflets (1.3–)2–4(–4.6) × (0.4–)1–1.7 cm, oblong-elliptic, apex rounded to sub-truncate, base obliquely rounded to sub-cordate, mid-rib nearly central, lamina sub-glabrous above, pubescent below. **Inflorescence**: an axillary spiciform raceme, 8–18 cm long, 1–3 per axil, peduncle and rachis pubescent. **Flowers**: greenish-white to yellow, pedicels 1–1.5 mm long; calyx 1–2 mm long, shallowly toothed, glabrous to slightly pubescent at teeth apices; petals 2.5–4 × 1–1.2 mm; stamen filaments 5–6 mm long. **Fruit**: a torulose, laterally compressed, slightly curved craspedium, 26–37 × 8–9 cm, with transverse septa between seeds dividing the fruit into one-seeded segments which, upon ripening, fall from the persistent replum; segments slightly umbonate over seeds. **Seeds**: ovoid, compressed, 1.2–1.5 × 0.9–1.1 × 0.3–0.4 cm, pleurogram heart-shaped, becoming diffuse near hilum.

### ﻿ var. bacillaris

Taxon classificationPlantaeFabalesFabaceae

E2316615-6702-5AAB-88A4-13367A77DCC8

= Entada nana Harms var. pubescens R.E. Fr., Schwed. Rhod.–Kongo–Exped. 1911–12, 1: 64. 1914.

#### Description.

Young stems with yellowish to golden hairs. Pinnae 3–4 pairs per leaf. Leaflets 8–13 pairs per pinna, (2–)2.5–4(–4.6) × (0.5–)1–1.6 cm. Calyx glabrous.

#### Distribution.

Zambia, southwest Tanzania.

#### Habitat and ecology.

Escarpment woodland with *Brachystegia*, *Julbernardia* Pellegr. and *Isoberlinia* Craib & Stapf (Lungu, 1995, p. 38), on shallow rocky soils; 900–1520 m alt.

### ﻿ var. plurijuga

Taxon classificationPlantaeFabalesFabaceae

Brenan, Kew Bull. 20(3): 372. 1966.

615FE426-97E0-5022-8BB8-6C1C24F4C487

#### Type.

ZAMBIA. Abercorn District, Inono Valley, 1 km from Mpulungu Road, *H.M. Richards 2278* (holotype: K [K000232133]).

#### Description.

Young stems and leaves with grey to golden hairs. Pinnae 3–10 pairs per leaf. Leaflets (10–)11–24 pairs per pinna, (1–)1.6–2.7 × 0.4–0.7 cm. Calyx sometimes sparsely hairy.

#### Distribution.

Zambia.

#### Habitat and ecology.

Similar to var. bacillaris, though Brenan (1970, p. 19) also noted var.plurijuga sometimes occurs on sandy soils; 1220-1740 m alt. Additionally, Lungu (1995, p. 38) stated that var. plurijuga has also been found on deep, well-drained soils on the edges of Miombo woodland and river valleys.

#### Note.

Brenan (1966, 1970) expressed uncertainty about the status and placement of this taxon, citing the possibility that it might represent a putative hybrid between var. bacillaris and *E.abyssinica* or be better placed as a variety of *E.chrysostachys*, stating that it differs from the latter only in its more numerous pinnae and longer stipe to the fruit (Brenan 1970, p. 19).

### ﻿ Entada borneensis

Taxon classificationPlantaeFabalesFabaceae

Ridl., J. Asiat. Soc. Bengal, Pt. 2, Nat. Hist. 67: 307. 1898.

2C960B75-E755-5238-8DF2-644DD6398952

#### Type.

MALAYSIA. Borneo, Sarawak, Sarawak River, Penkulu Ampat, *G.D. Haviland s.n.* (holotype: K [K000635744]).

#### Description.

Liana > 40 m long. **Leaves**: rachis 5–9.5 cm, sub-glabrous to tomentose, terminating in a bifurcating tendril; pinnae 2 opposite pairs per leaf, each with 3–7 pairs of alternate to sub-opposite leaflets, except for the distal opposite pair; leaflets narrowly oblong to obovate, 1.4–4 × 0.7–1.8 cm, base asymmetrically rounded, apex rounded and emarginate, both surfaces glabrous, main vein puberulous. **Inflorescence**: a 19–40 cm long, solitary, axillary spike, rachis tomentose. **Flowers**: yellowish or greenish-white to white, sessile, staminate or bisexual; calyx cupular, 0.5–0.6 mm long, glabrous; petals 2 × 0.6–0.8 mm; stamen filaments 4–6 mm long. **Fruit**: a gigantic, torulose craspedium, 50–120 × 10–13 cm, with transverse septa between seeds dividing the fruit into one-seeded segments which, upon ripening, fall from the persistent replum; segments 9–10 cm long; epicarp coriaceous, endocarp chartaceous. **Seeds**: circular, laterally compressed, 4 cm in diameter, hard, brown, lacking a pleurogram.

#### Distribution.

Borneo.

#### Habitat and ecology.

Primary and secondary rainforest, especially along rivers; in sandy clay substrates, loams and soils derived from limestone; 0–800 m alt.

#### Note.

Nielsen (1992) noted that *E.borneensis* is locally common but rarely collected.

### ﻿ Entada burkei

Taxon classificationPlantaeFabalesFabaceae

(Benth.) S.A. O’Donnell & G.P. Lewis comb. nov.

D702A30B-6E73-5077-8C40-BBF5775B0D2A

urn:lsid:ipni.org:names:77303568-1

#### Type.

SOUTH AFRICA. Transvaal, Magaliesberg, *Burke & Zeyher s.n.* (holotype: K [K000232271]; presumed isotypes (fide Ross 1975a: p. 144): BM [BM000842178], MO [MO-954355], TCD, Z).

#### Basionym.

*Elephantorrhizaburkei* Benth., London J. Bot. 5: 81. 1846.

#### Description.

**Figure 9.**
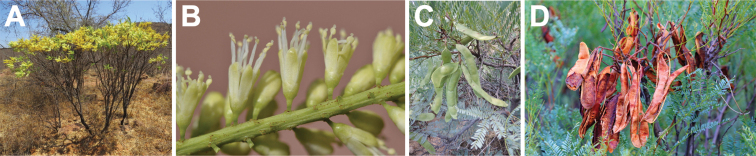
*Entadaburkei* habit and reproductive structures. **A** branched shrub bearing leaves of the current season’s growth alongside inflorescences near shoot tips, South Africa (photo: J Heymans *CC BY-NC-ND 4.0*, iNaturalist (2021) observation 11078242) **B** open, pedicellate flowers with minute red glands at base of pedicels, South Africa (photo: J-H Keet *CC BY-NC 4.0*, Ueda (2021) observation 11099684) **C** leaves and immature fruits, South Africa (photo: M Schmidt, Dressler et al. (2014a)) **D** leaves and mature fruits, South Africa (photo: tjeerd *CC BY-NC 4.0*, Ueda (2021) observation 38383810).

Shrub to small tree (0.3–)1–3(–6) m, with dark grey to reddish bark (Figs 2C, 9A). **Leaves**: petiole 2.6–6.5 cm long; rachis 3.6–14.5 cm long; pinnae (1–)4–8(–9) pairs, 3.5–12.5 m long, with (9–)12–23(–32) pairs of leaflets; leaflets 7–17 × 1.5–3.5(–5) mm, oblanceolate to elliptic or linear-oblong, apex obtuse to rounded, base slightly oblique, lamina glabrous. **Inflorescence**: an axillary spiciform raceme borne on lateral shoots of the current season’s growth, 5–10(–12) cm long, solitary or aggregated in fascicles, rachises glabrous. **Flowers**: cream, yellow or yellowish-white, pedicels 2 mm long, articulated near the middle, with minute reddish glands at the base of the pedicels; calyx campanulate, 2.5 mm long, distinctly toothed, glabrous; petals 3–4.5 mm long; stamen filaments 5 mm long (Fig. 9B). **Fruit**: a laterally compressed, straight to slightly curved craspedium, 10–19(–28) × 2.5–4 cm, transverse veins prominent, lacking transverse septa between seeds, the valves separating from the replum intact upon ripening, the epicarp exfoliating from the endocarp (Figs 2M, 9C, D). **Seeds**: irregular in shape, 9–13 × 8–12 × 8 mm.

#### Distribution.

Botswana, Zimbabwe, Mozambique, South Africa.

#### Habitat and ecology.

Woodland, grassland and scrub, usually in rocky settings; 970–1370 m alt.

### ﻿ Entada camerunensis

Taxon classificationPlantaeFabalesFabaceae

Villiers, Bull. Mus. Natl. Hist. Nat., B, Adansonia 4: 193. 1983.

7F8EFC02-1B0C-526B-9774-08A720752A54

#### Type.

CAMEROON. West Kongolo, on bank of River Bayo, *R. Letouzey 3534* (holotype: P [P00418283, P00418284 & P00418285]; isotype: YA [YA0023378]).

#### Description.

Liana, sometimes sarmentose, stem twisted, to 15 cm diameter at base. **Leaves**: a conspicuous ridge at petiole base; rachis 5.5–7.9(–9.5) cm, grooved above, tendrils absent, but petioles sometimes modified for climbing; pinnae 2–4 pairs per leaf, 3.5–10(–16) cm long with 5–10 pairs of leaflets; leaflets 1–2.5 × 0.3–1.1 cm, obovate-oblong, increasing in size distally, apex truncate to retuse, base asymmetric with proximal margin rounded, distal margin attenuate, lamina pubescent. **Inflorescence**: a terminal or axillary spiciform raceme, 7–9.5 cm long, solitary or 2 per axil, peduncle and rachis pubescent. **Flowers**: yellow to greenish-yellow, staminate or bisexual, pedicels 0.5–0.75 mm long; calyx cupular, 0.75–1.25 mm long, shallowly toothed, glabrous to sparsely pubescent at tooth apices; petals 3–3.25 × 0.6–0.8 mm, elliptic to obovate; stamen filaments 3–5 mm long. **Fruit**: a torulose, laterally compressed, slightly curved craspedium, 20–29 × 7–9 cm, with transverse septa between seeds dividing the fruit into one-seeded segments which, upon ripening, fall from the persistent replum; segments distinctly umbonate over seeds. **Seeds**: elliptic-oblong, laterally compressed, 1.7–1.9 × 0.9–1 cm, pleurogram open.

#### Distribution.

Cameroon, Democratic Republic of Congo, Zambia.

#### Habitat and ecology.

Riparian forests.

### ﻿ Entada chrysostachys

Taxon classificationPlantaeFabalesFabaceae

(Benth.) Drake, Hist. Phys. Madagascar 30: 51. 1902.

6E465CCF-9DEF-53CB-A3D5-F320A3C4D078

= Entada kirkii Oliv., Fl. Trop. Afr. 2: 327. 1871.
= Entada boiviniana (Baill.) Drake, A. Grandidier, Hist. Phys. Madagascar 30: 51. 1902. (publ. 1903).
= Entada grandidieri (Baill.) Drake, A. Grandidier, Hist. Phys. Madagascar 30: 51. 1902. (publ. 1903).

#### Type.

MADAGASCAR. Emirna Province [Imerina] and Imamou, *W. Bojer s.n.* (holotype: K; isotypes: M [M0218663], P [P00367635 & P00367637]).

#### Basionym.

*Adenantherachrysostachys* Benth., J. Bot. (Hooker) 4: 343. 1841.

#### Description.

**Figure 10.**
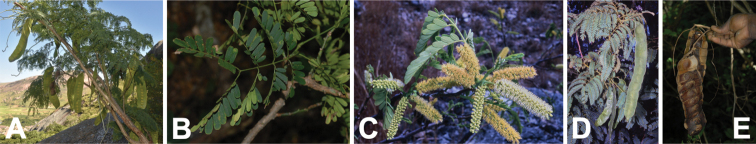
*Entadachrysostachys* habit, vegetative and reproductive structures. **A** small tree, Madagascar (photo: thierrycordenos *CC BY-NC 4.0*, Ueda (2021) observation 25108890) **B** leaf, Mozambique (photo: O Maurin *CC BY-NC-SA 3.0*, iBOL (2016) record SAFH1507–11) **C** axillary spiciform racemes of open, pedicellate flowers and closed flower buds, Madagascar (photo: D Du Puy) **D** leaves and immature pods, Madagascar (photo: D Du Puy) **E** mature pods, Mozambique (photo: O Maurin *CC BY-NC-SA 3.0*, iBOL (2016) record SAFH1507–11).

Shrub or small tree to 10 m tall or liana to 12 m, stem to 20 cm thick, often twisted (Fig. 10A). **Leaves**: rachis 8–16 cm long, grooved above, glabrous; pinnae (2–)3–5(–8) pairs per leaf, (4.5–)5.2–8.9(–13) cm long, with 10–17(–21) pairs of leaflets; leaflets 13–19(–29) × (3–)4.9–5.5(–10) mm, oblong to obovate-oblong, apex rounded, base asymmetric rounded on proximal margin and cuneate on distal margin, mid-rib diagonal and raised above and below, lamina appressed-pubescent to glabrous (Fig. 10B). **Inflorescence**: an axillary spiciform raceme, 4–12(–13.5) cm long, usually clustered, but sometimes solitary, rachis pubescent or glabrous (Fig. 10C). **Flowers**: white to yellow, pedicels 1–1.5 mm long, with an unpleasant odour; calyx obconical, 1–1.5 mm long, glabrous to sparsely pubescent, distinctly toothed; petals 3–4 × 1–1.4 mm; stamen filaments 4–6 mm long. **Fruit**: a torulose, laterally compressed, slightly curved craspedium 20–45 × 5–10 cm, with transverse septa between seeds dividing the fruit into one-seeded segments which, upon ripening, fall from the persistent replum (Fig. 10D, E). **Seeds**: elliptic, 14–17.7 × 10.5–12.8 × 3.3–4 mm, pleurogram elliptic, open near hilum.

#### Distribution.

Madagascar, Comoro Islands, Mozambique, Zimbabwe, Zambia, Malawi, Tanzania.

#### Habitat and ecology.

Disturbed forests and grassland; riparian thicket; woodland characterised by *Brachystegiaglaucescens* Hutch. & Burtt Davy; and seasonally wet valley bottoms with *Combretum*; sandy soils and laterite.

### ﻿ Entada dolichorrhachis

Taxon classificationPlantaeFabalesFabaceae

Brenan, Kew Bull. 20: 374. 1966. (publ. Jan. 1967).

2C627F9F-422E-5819-B1C3-AD33D9D31F16

#### Type.

ZAMBIA. Mbala (Abercorn) District, Lufubu River, Iyendwe Valley, on path to Shulu Kwesa Village, *H.M. Richards 11952* (holotype: K; isotypes: BR [BR0000006251536], LISC [LISC001666], NY [NY00002026], SRGH).

#### Description.

**Figure 11.**
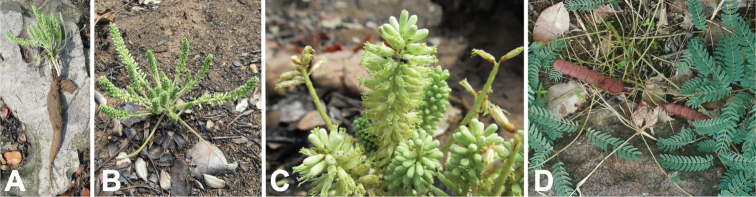
*Entadadolichorrhachis* habit, reproductive and vegetative structures. **A** uprooted geoxylic suffrutex, Zambia (photo: M Bingham, Bingham et al. (2021)) **B** spiciform racemes in axils of short, erect shoot with two young leaves expanding towards base of image, Zambia (photo: M Bingham, Bingham et al. (2021)) **C** open and unopened flowers, Zambia (photo: M Bingham, Bingham et al. (2021)) **D** mature fruits and trailing, scandent leaves with elongate rachises, Zambia (M Bingham, Bingham et al. (2021)).

Geoxylic suffrutex with erect annual stems, 1–10 cm tall, young shoots pubescent (Figs 2D, 11A). **Leaves**: elongate and trailing on the ground; rachis (15–)42–65(–90) cm long, expanding from the apex during the growing season, tendrils lacking; pinnae 19–35 pairs on mature leaves, 2.7–5 cm long, with 6–9(–16) pairs of leaflets; leaflets (5–)8–17(–20) × 2.5–9.3 mm, ovate-oblong, asymmetric, apex rounded and mucronate, base oblique, lamina glabrous above, pubescent below (Fig. 11D). **Inflorescence**: an axillary spiciform raceme, 3.4–10 cm long, 1–2 per axil, rachis densely pubescent (Fig. 11B). **Flowers**: greenish-yellow, pedicels 1.5–2.5 mm long; calyx 1.5–2 mm long, deeply toothed, pubescent; petals pale dull yellow, 4.75–5.75 mm long; stamen filaments 7–10 mm long (Fig. 11C). **Fruit**: a torulose, straight craspedium, 3–6.5 × 1.5–1.8 cm, with transverse septa between seeds dividing the fruit into one-seeded segments which, upon ripening, fall from the persistent replum; segments umbonate over seeds (Fig. 11D). **Seeds**: ovate, laterally compressed, 9 × 8 mm, with open pleurogram.

#### Distribution.

Zambia.

#### Habitat and ecology.

Woodland and open riverbanks, on sandy soil; 780–1620 m alt.

### ﻿ Entada elephantina

Taxon classificationPlantaeFabalesFabaceae

(Burch.) S.A. O’Donnell & G.P. Lewis comb. nov.

28C1E326-7269-59F7-8F8F-03FBE2464E11

urn:lsid:ipni.org:names:77303964-1

≡ Elephantorrhiza elephantina (Burch.) Skeels, Bull. Bur. Pl. Industr. U.S.D.A. 176: 29. 1910.

#### Type.

SOUTH AFRICA. Cape Province, Bechuland Division, Kuruman District, between Matlowing River and Kuru, *W.J. Burchell 2410* (holotype: K [K000232273]; isotypes: GH [GH00058379], P [P00418275]).

#### Basionym.

*Acaciaelephantina* Burch., Trav. S. Africa 2: 236. 1824.

#### Description.

**Figure 12.**
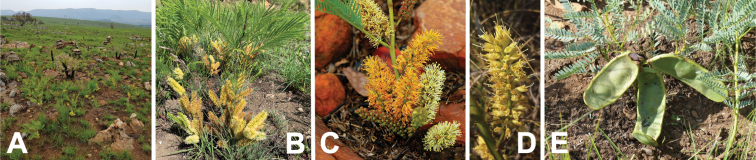
*Entadaelephantina* habitat, habit, reproductive and vegetative structures. **A** growing in fire-prone open scrub, South Africa (photo: R Gill *CC BY-NC 4.0*, Ueda (2021) observation 32241269) **B** geoxylic suffrutex with erect herbaceous shoots bearing finely divided bipinnate leaves and producing spiciform racemes near ground level, Eswatini (photo: L Loffler *CC BY-NC 4.0*, Ueda (2021) observation 44861491) **C** spiciform racemes clustered near the base of the stem, South Africa (photo: tjeerd *CC BY-NC 4.0*, Ueda (2021) observation 62025284) **D** spiciform raceme of open flowers, South Africa (photo: J Whatmore, Ueda (2021) observation 62547631) **E** immature fruits borne near the base of the stem, South Africa (photo: G Lewis).

Geoxylic suffrutex with erect, annual, herbaceous stems 20–90 cm arising from the woody end of an elongate subterranean axis (Fig. 12A, B). **Leaves**: petiole 1.3–3.6(–8) cm long, rachis 3.5–13.5(–17.5) long; pinnae 2–4 pairs on lower leaves, 7–17 pairs on upper leaves, 3–9(–10.5) cm long, with (7–)12–45(–55) pairs of leaflets; leaflets (4–)5–10(–15) × (0.3–)0.5–2(–2.5) mm, linear to linear-oblong, apex acute to rarely obtuse, sometimes asymmetric, mucronate, base oblique, lamina glabrous (Fig. 12B, E). **Inflorescence**: an axillary spiciform raceme usually confined to the lower part of the stem, (2–)4–8(–12) cm long, solitary or grouped, rachises usually glabrous (Fig. 12B–D). **Flowers**: cream-coloured, yellow or yellowish-white, pedicels 1.5 mm long, articulated near the middle, with minute reddish to reddish-brown glands at the base; calyx campanulate, 1.75 mm long, distinctly toothed, glabrous; petals 2.75–3.75 mm long; stamen filaments 6.5 mm long (Fig. 12D). **Fruit**: a laterally compressed, straight to slightly curved craspedium, (5–)9.5–15(–21) × 3–5.7 cm, lacking transverse septa between seeds, thus leaving the valves to separate from the replum intact upon ripening, the epicarp exfoliating from the endocarp; umbonate over seeds (Fig. 12E). **Seeds**: ellipsoid, 18–26 × 13–18 × 6–13 mm.

#### Distribution.

Namibia, Botswana, Zimbabwe, Mozambique, South Africa, Eswatini, Lesotho.

#### Habitat and ecology.

Grassland and open scrub, sometimes gregarious (Fig. 12A); 1060–1360 m alt.

#### Note.

Brenan (1970, p. 28) and Ross (1974, p. 250; 1975a, p. 141) noted that leaf characters vary considerably across the range of *E.elephantina*. Specimens from the western portion of the range tend to have fewer pinnae and leaflets with larger leaflets; those from eastern areas bear more numerous pinnae and leaflets, with smaller leaflets. This variation appears to be continuous, so neither author attempted to subdivide the taxon.

### ﻿ Entada gigas

Taxon classificationPlantaeFabalesFabaceae

(L.) Fawc. & Rendle, Fl. Jamaica 4: 124. 1920.

D340841A-FA53-5F19-877E-363A3EA9DD75

= Entada gigalobium DC., Mém. Légum.: 421. 1826.
= Entada scandens (L.) Benth. subsp. planoseminata De Wild., Pl. Bequaert. 3: 85. 1925.
= Entada scandens (L.) Benth. subsp. umbonata De Wild., Pl. Bequaert. 3: 86. 1925.
= Entada planoseminata (De Wild.) G.C.C. Gilbert & Boutique, Fl. Congo Belge 3: 221. 1952.
= Entada umbonata (De Wild.) G.C.C. Gilbert & Boutique, Fl. Congo Belge 3: 222. 1952.

#### Type.

SWEDEN (cultivated). Uppsala Botanic Garden, *Herb. Linn. No. 1228.11* (neotype: LINN, designated by Panigrahi in Taxon 34: 714. 1985).

#### Basionym.

*Mimosagigas* L., Fl. Jamaic. (Linnaeus) 22. 1759.

#### Description.

**Figure 13.**
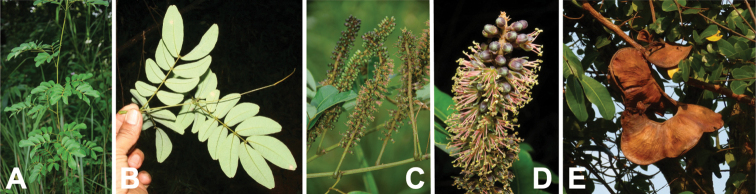
*Entadagigas* habit, vegetative and reproductive structure. **A** vegetative shoot of liana, Gabon (photo: E Bidault *CC BY-NC-ND 3.0*, MBG (2021)) **B** abaxial view of a leaf, Costa Rica (photo: D Janzen *CC BY-ND-SA 3.0*, iBOL (2016) record MHPAD2192–10) **C** axillary spiciform racemes, Gabon (photo: E Bidault *CC BY-NC-ND 3.0*, MBG (2021)) **D** open, pedicellate flowers, Gabon (photo: E Bidault *CC BY-NC-ND 3.0*, MBG (2021)) **E** large, mature fruit twisted into a lax spiral, Democratic Republic of Congo (photo: W McCleland, Dressler et al. (2014a)).

Liana to 45 m long (Fig. 13A). **Leaves**: rachis 5.9–7.5 cm long, terminating in a bifurcating tendril; pinnae (1–)2 pairs per leaf, with (3–)4(–5) pairs of leaflets; leaflets oblong to elliptic, often asymmetric, apex obtuse or rounded, emarginate, both surfaces of lamina essentially glabrous, except beneath near the base and the mid-rib puberulous above and sometimes below (Fig. 13A, B). **Inflorescence**: a spiciform raceme, 8–25 cm long, solitary, supra-axillary (3–5 mm above the axil) with tufted glands between the axil and point of insertion of the rachis, ± pubescent, peduncle 1.5–6 cm long (Fig. 13C). **Flowers**: creamy white to greenish-yellow, pedicels 1–1.5 mm long; calyx 1–1.25 mm long, glabrous to pubescent; petals 2.5–3 mm long; stamen filaments 3.5–6 mm long (Fig. 13E). **Fruit**: a gigantic craspedium, 40–120 × 7.5–12 cm, less woody than in the morphologically similar *E.rheedei*, twisted into a lax spiral, with transverse septa between seeds dividing the fruit into one-seeded segments which, upon ripening, fall from the persistent replum; epicarp falling away to expose an inflexible chartaceous endocarp; 10–12-seeded (Figs 2K, 13E). **Seeds**: circular to slightly cordate, laterally compressed, 4–5.5 cm in diameter, hard; cotyledons separated by an intervening air space, enabling flotation (Fig. 2O).

#### Distribution.

Central and west Africa; Central America, Caribbean and Colombia.

#### Habitat and ecology.

Riparian forests; Brenan (1959) noted two specimens collected from Uganda (*Jarrett 400*; *Brown 328*) at 1310 m alt. and 1183 m alt., respectively. Seeds dispersed widely by sea currents.

### ﻿ Entada glandulosa

Taxon classificationPlantaeFabalesFabaceae

Pierre ex. Gagnep., Notul. Syst. (Paris) 2: 57. 1911.

D586FBFD-8FD9-519C-B171-49AB6E6A1A9A

= Entada tamarindifolia Pierre ex. Gagnep., Notul. Syst. (Paris) 2: 59. 1911.

#### Type.

LAOS. *Massie s.n.* (lectotype: P [P02436137], designated by I.C. Nielsen in Adansonia ser. 2, 19: 342. 1980).

#### Description.

**Figure 14.**
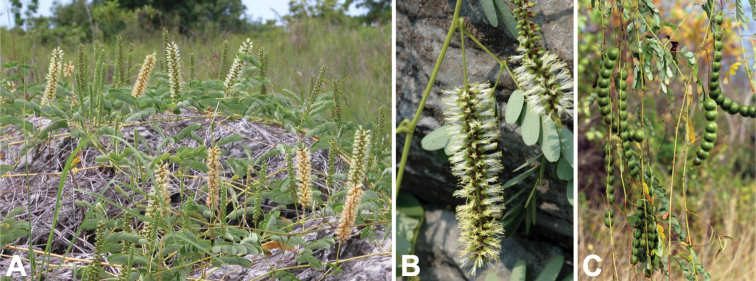
*Entadaglandulosa* habit and reproductive structures. **A** scandent shrub with erect axillary spikes, Thailand (photo: T Boonkerd, all rights reserved) **B** solitary axillary spikes with open flowers, Thailand (photo: T Boonkerd, all rights reserved) **C** immature fruits, Thailand (photo: T Boonkerd, all rights reserved).

Shrub, scandent (Fig. 14A). **Leaves**: petiole 1.8–4 cm long, rachis 4.5–10 cm long, terminating in a bifurcating tendril; pinnae 2 pairs pair leaf, 4–8 cm long, with 5–6 pairs of leaflets; leaflets 1.1–4 × 0.5–1.7 cm, elliptic to oblong, base truncate, apex emarginate or mucronate. **Inflorescence**: a spike 7–18 cm long, axillary, solitary, rachis pubescent to velutinous (Fig. 14A, B). **Flowers**: creamy white to yellowish-white, sub-sessile; calyx cupular, 2–2.5 mm long, glabrous to puberulous; petals lanceolate, 5 × 1 mm, a pair of linear glands on the lower half of the dorsal side of each petal; stamen filaments 8 mm long (Fig. 14B). **Fruit**: a torulose, curved craspedium, 35 × 2.2–2.6 cm, with transverse septa between seeds dividing the fruit into one-seeded segments which, upon ripening, fall from the persistent replum; segments 2.4 cm long; epicarp coriaceous, endocarp papyraceous (Fig. 14C). **Seeds**: sub-globular, 1.1–1.8 cm, hard, brown, pleurogram lacking.

#### Distribution.

Laos, Cambodia, southern Vietnam, Thailand, Myanmar.

#### Habitat and ecology.

Seasonally dry deciduous forest, mixed forest with Dipterocarpaceae and evergreen forest, up to 500 m alt. Usually on limestone, though also in shallow sandy soils and in red soils.

### ﻿ Entada goetzei

Taxon classificationPlantaeFabalesFabaceae

(Harms) S.A. O’Donnell & G.P. Lewis comb. nov.

EA5556BF-388A-5284-9FAE-57A4EC3C32D1

urn:lsid:ipni.org:names:77303569-1

≡ Elephantorrhiza goetzei (Harms) Harms, Veg. Erde [Engler] 9(3, 1): 400, in obs. 1915.

#### Type.

TANZANIA. Rufiji District, *W. Goetze 82* (holotype: B†; drawing: BM [BM000842177]; isotype: K).

#### Basionym.

*Piptadeniagoetzei* Harms, Bot. Jahrb. Syst. 28: 397. 1900.

#### Description.

**Figure 15.**
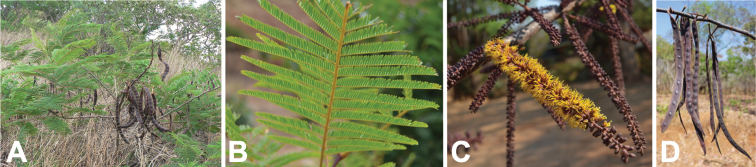
*Entadagoetzei* habit, vegetative and reproductive structures. **A** shrub with mature fruits, Malawi (photo: G Baumann, Dressler et al. (2014a)) **B** leaf with linear-oblong leaflets, Malawi (photo: C Chisale, Dressler et al. (2014a)) **C** fascicles of spiciform racemes bearing open, pedicellate flowers and closed flower buds on leafless stem, Malawi (photo: C Chisale, Dressler et al. (2014a)) **D** mature fruits, Malawi (photo: C Chisale, Dressler et al. (2014a)).

Shrub to small deciduous tree 1–4(–7) m tall, young shoots often becoming blackish (Fig. 15A). **Leaves**: petiole 1–5(–7.5) cm; rachis 6–20(–45.5) cm, grooved above; pinnae 3–30(–41) pairs per leaf, 1.8–9 cm long, with 9–40(–48) pairs of leaflets; leaflets 3.5–12(–22) × 0.7–0.8(–2.75) mm, linear-oblong to narrowly oblong, apex acute to rounded and mucronate, base oblique, mid-rib running from distal corner of leaflet base to apex centre, lamina glabrous (Fig. 15B). **Inflorescence**: a spiciform raceme, (2–)5–20(–23) cm long, axillary, solitary or aggregated in fascicles or on short lateral shoots, rachis glabrous (Figs 2H, 15C). **Flowers**: yellowish-white, sometimes tinged pink or purple, pedicels 1 mm, articulated near the middle, with minute pale yellowish-white glands at the base of the pedicels; calyx 1.5–1.75 mm long, distinctly toothed, glabrous; petals 2.5–3 mm long; stamen filaments 4.5 mm long (Fig. 15C). **Fruit**: a straight to curved craspedium, (15–)20–30(–44) × 1.3–2.2(–3) cm, lacking transverse septa between seeds, thus leaving the valves to separate from the replum intact upon ripening, the epicarp exfoliating from the endocarp; umbonate over seeds (Fig. 15D). **Seeds**: ellipsoid to lenticular, 11–20 × 9–18 × 7–12 mm.

### ﻿ subsp. goetzei

Taxon classificationPlantaeFabalesFabaceae

F7AA30F8-4910-5098-9DAD-36C47B278304

#### Description.

Leaves with (3–)14–41 pairs of pinnae per leaf, pinna rachis 3.5–9.5 cm long. Leaflets (11–) 20–48 pairs per pinna, 3.5–12 × 0.7–3 mm.

#### Distribution.

Tanzania, Angola, Botswana, Zambia, Zimbabwe, Malawi, Mozambique, South Africa (Transvaal).

#### Habitat and ecology.

Woodland and scrub, usually on rocky substrates, but also on alluvial soils; 120–1460 m alt.

#### Note.

Ross (1974, 1975a) noted that plants from the area delimited for Flora Zambesiaca (e.g. those referred to in Brenan 1970, p. 26) frequently flower when the plant is leafless, whereas those from the Transvaal produce flowers together with leaves.

### ﻿ subsp. lata

Taxon classificationPlantaeFabalesFabaceae

(Brenan & Brummitt) S.A. O’Donnell & G.P. Lewis comb. nov.

2798AEC1-BFA2-5239-B29E-1017656C3549

urn:lsid:ipni.org:names:77303965-1

#### Type.

ZAMBIA. Katombora, *Morze 55* (holotype: FHO [00096339U]).

#### Basionym.

Elephantorrhizagoetzei(Harms)Harmssubsp.lata Brenan & Brummitt, Bol. Soc. Brot., Sér. 2, 39: 189. 1965.

#### Description.

Leaves with 4–15 pairs of pinnae, pinna rachis 6.5–15 cm long. Leaflets 9–28 pairs per pinna, 12–22 × 4–8 mm.

#### Distribution.

Zambia, Zimbabwe.

#### Habitat and ecology.

Woodland of various types.

#### Note.

Grobler (2012, p. 129) does not accept subspecific taxa within *E.goetzei* on the basis that the additional material she collected across the species range revealed the morphological variation in leaf characters to be continuous.

### ﻿ Entada hockii

Taxon classificationPlantaeFabalesFabaceae

De Wild., Repert. Spec. Nov. Regni Veg. 11: 535. 1913.

44F08734-2098-54F4-9A4E-652D14C59A96

#### Type.

DEMOCRATIC REPUBLIC OF CONGO. Haut–Katanga, Plateau de la Manika, *A. Hock s.n.* (holotype: BR [BR0000008916471]).

#### Description.

Geoxylic suffrutex, annual stems pubescent. **Leaves**: rachis 6–9.7 cm long, pubescent; pinnae 1–2 pairs per leaf, 6–8.1 cm long, with 7–9 pairs of leaflets; leaflets 1.4–2.3 × 0.65–0.85 cm, oblong, apex obtuse to rounded, base obtuse to sub-truncate, lamina glabrous above, pubescent below. **Inflorescence**: an axillary, spiciform raceme 3.5–7 cm long, 1–3 per axil, rachis densely pubescent. **Flowers**: cream-coloured, pedicels 0.5–1 mm long; calyx 1 mm long, distinctly toothed, glabrous; petals 2.8–3.4 × 1–1.3 mm; stamen filaments 2.8–3 mm long. **Fruits and seeds**: not seen.

#### Distribution.

Democratic Republic of Congo, Angola.

#### Habitat and ecology.

On Kalahari sands.

### ﻿ Entada leptostachya

Taxon classificationPlantaeFabalesFabaceae

Harms, Bot. Jahrb. Syst. 53: 456. 1915.

ADDF20A4-18EA-57D0-A9B4-15068290A422

#### Type.

KENYA. Machakos District, Kibwezi, *G. Scheffler 120* (lectotype: P [P00418289], designated by J.-F. Villiers in Leguminosae of Madagascar, 2002: 165; isolectotype: K [K000232161]; original syntype: B†).

#### Description.

Liana, shrub or small tree, 3–6 m, stems twining, with elevated nectaries at nodes (Fig. 4A). **Leaves**: rachis (4.5–)5.6–15.1(–16) cm long, tendrils absent, but plant climbing using modified, hooked pinnae on long shoots; pinnae 2–4(–5) pairs per leaf, (4–)5.6–6.8(–13) cm long, with 7–11(–14) pairs of leaflets; leaflets 9–25(–35) × 3–9(–15) mm, oblong to oblanceolate-oblong, apex rounded to emarginate, base asymmetric, lamina usually puberulous above and below though sometimes sub-glabrous to glabrous. **Inflorescence**: an axillary spike, 3–8(–16) cm long, 1–3 per axil together on short shoots, rachis glabrous. **Flowers**: yellow, sweetly scented; calyx obconical, 0.5–1 mm long, shallowly toothed, glabrous; petals 2–2.5 × 0.8 mm; stamen filaments 2.5–4 mm long. **Fruit**: a torulose, laterally compressed craspedium, 17–23 × 4.3–8.4 cm, with transverse septa between seeds dividing the fruit into one-seeded segments which, upon ripening, fall from the persistent replum. **Seeds**: elliptic, 10.4–14 × 9–10.6 × 3.5–3.7 mm, pleurogram oval, open.

#### Distribution.

Ethiopia, Somalia, Kenya, Tanzania, Madagascar.

#### Habitat and ecology.

Dry scrub, degraded woodland with scattered trees, dense *Commiphora* Jacq. Woodland; growing as small trees when on steep limestone slopes.

### ﻿ Entada louvelii

Taxon classificationPlantaeFabalesFabaceae

(R. Vig.) Brenan, Kew Bull. 20: 365. 1966.

A06F1033-675A-535D-8FB2-5D9AFA0431E2

#### Type.

MADAGASCAR. Analamazoatra, south of Moramanga, *M. Louvel 16* (lectotype: P [P00452896], designated by J.-F. Villiers in Leguminosae of Madagascar, 2002: 165).

#### Basionym.

Entadapervillei (Vatke) R. Vig. var. louvelii R. Vig., Notul. Syst. (Paris) 13: 347. 1949.

#### Description.

**Figure 16.**
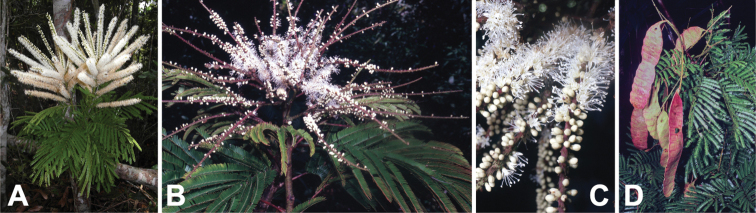
*Entadalouvelii* vegetative and reproductive structures. **A** branch and shoots bearing leaves and terminal panicles of spikes, Madagascar (photo: P Antilahimena *CC BY-NC-ND 3.0*, MBG (2021)) **B** terminal panicle of flower spikes, Madagascar (photo: D Du Puy) **C** spikes bearing open, sessile flowers and closed flower buds, Madagascar (photo: D Du Puy) **D** leaves and fruits nearing maturity, Madagascar (photo: D Du Puy).

Tree 10–15 m tall, with elevated nectaries at nodes (Fig. 4B). **Leaves**: petiole 2–4 cm long, grooved above; rachis 9–18 cm long, winged, no tendril; pinnae 11–20 pairs per leaf, 3–9 cm long, with 24–46 pairs of leaflets; leaflets 3–7 × 1–1.75 mm, oblong to oblong-elliptic, apex rounded-obtuse to sub-acute and mucronate, base asymmetric and sub-truncate; lamina glabrous (Fig. 16A, B, D). **Inflorescence**: a terminal panicle of spikes, each spike 5–19 cm long, rachis pubescent (Fig. 16A, B). **Flowers**: white, 4–5 mm long, sessile to sub-sessile; calyx cream-coloured, obconical, 1.5–2 mm long, shallowly toothed, glabrous; petals 3.5–4 mm long; stamen filaments 5–7.25 mm long (Fig. 16C). **Fruit**: a torulose, laterally compressed craspedium, 15–20 × 3–6.5 cm, with transverse septa between seeds dividing the fruit into one-seeded segments which, upon ripening, fall from the persistent replum (Fig. 16D). **Seeds**: elliptic, 1.5–2.4 × 0.6–1.2 cm, light brown, pleurogram lacking.

#### Distribution.

Madagascar (east).

#### Habitat and ecology.

Moist forest, up to 1000 m alt. (Villiers 2002, p. 167); disturbed or dry forest (Lungu 1995).

### ﻿ Entada mannii

Taxon classificationPlantaeFabalesFabaceae

(Oliv.) Tisser., Bull. Soc. Bot. France 99: 257. 1953.

911D01B2-83D2-5140-B3A4-B31691427521

= Entada bequaertii De Wild., Pl. Bequaert. 3: 79. 1925.

#### Type.

EQUATORIAL GUINEA. Fernando Pó (Boiko), *Mann 414* (holotype: K [K000232169]).

#### Basionym.

*Piptadeniamannii* Oliv., Fl. Trop. Afr. [Oliver et al.] 2: 329. 1871.

#### Description.

**Figure 17.**
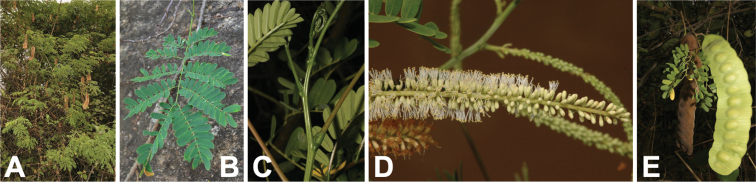
*Entadamannii* habit, vegetative and reproductive structures. **A** scandent shrub with mature fruits, Mali (photo: P Birnbaum, Dressler et al. (2014a)) **B** leaf bearing oblong leaflets, Malawi (photo: C Chisale, Dressler et al. (2014a)) **C** young shoot with swollen nodes either side of petiole insertion, Mali (photo: P Birnbaum, Dressler et al. (2014a)) **D** spiciform raceme with open, pedicellate flowers and closed flower buds, Congo (photo: D Harris, Dressler et al. (2014a)) **E** leaf and nearly mature fruits, Mali (photo: P Birnbaum, Dressler et al. (2014a)).

Shrub, scandent, sometimes becoming arborescent, to 30 m, stem 15 cm diameter near base, glabrous (Fig. 17A). **Leaves**: rachis 5–20 cm long, sparsely pubescent; pinnae 3–6 pairs per leaf, one or more pinnae sometimes modified into a tendril, leaflet-bearing pinnae 4–6 cm long, with 8–13 pairs of leaflets; leaflets 4–16(–21) × 1.5–7 mm, oblong, apex retuse, base rounded, asymmetric, lamina glabrous to puberulous above, pubescent below (Fig. 17B). **Inflorescence**: an axillary spiciform raceme, 5.5–10 cm long, in panicles from the upper axils, rachis pubescent (Fig. 17D). **Flowers**: white, minutely pedicellate; calyx 0.7–1 mm, shallowly toothed, glabrous to puberulous; petals 2 mm long (Fig. 17D). **Fruit**: a torulose, laterally compressed, straight craspedium, 15–45 × 6–10 cm, with transverse septa between seeds dividing the fruit into one-seeded segments which, upon ripening, fall from the persistent replum (Fig. 17E). **Seeds**: elliptic, 1.8 × 0.9 cm, pleurogram present.

#### Distribution.

Tropical West Africa, from Senegal to Angola.

#### Habitat and ecology.

Riparian forest and on rocky hills in forest.

### ﻿ Entada mossambicensis

Taxon classificationPlantaeFabalesFabaceae

Torre, Contr. Conhec. Fl. Mocamb. 2: 88. 1954.

14609BDE-14E9-56F9-8150-F57CCFEC20C7

#### Type.

MOZAMBIQUE. Niassa, Nampula, *A.R. Torre 4750 A* (holotype: LISC [LISC001696, LISC001697, LISC001698, LISC001699]; isotypes: BM, K [K000232129, K000232130], BR [BR0000006251864]).

#### Description.

Shrub, sub-erect, 1–2 m tall, roots thick, fusiform. **Leaves**: rachis 6.7–8 cm long, tendrils lacking; pinnae 3–7 pairs per leaf, 7–9 cm long, with (40–)138–154 pairs of leaflets; leaflets 2.1–5.5 × 0.4–0.8 mm, linear-oblong, apex sub-acute and mucronate, base asymmetric, lamina glabrous. **Inflorescence**: an axillary spiciform raceme, 12–30 cm long, solitary. **Flowers**: purple, pedicels 2–2.5 mm long; calyx 1 mm long, glabrous; petals 4.5–5 mm long; stamen filaments 5–6 mm long. **Fruit**: a torulose, laterally compressed, falcate craspedium, 10–12 × 2–2.5 cm, with transverse septa between seeds dividing the fruit into one-seeded segments which, upon ripening, fall from the persistent replum. **Seeds**: 1.2 × 1 cm, 2.3 mm thick, with closed pleurogram.

#### Distribution.

Mozambique.

#### Habitat and ecology.

Rocky habitats.

### ﻿ Entada nudiflora

Taxon classificationPlantaeFabalesFabaceae

Brenan, Kew Bull. 20: 377. 1966. (publ. Jan. 1967).

ACA37B56-C67D-5A16-B750-E887828B8D10

#### Type.

ZAMBIA. Mbala (Abercorn) District, path to Kapata village, *H.M. Richards 10192* (holotype: K [K000232154, K000232155]).

#### Description.

Climber, slender, woody, up to 3 m. **Leaves**: rachis 4–6 cm long, terminating in bifurcating tendril or the petiolules of the terminal pinna pair modified for coiling; pinnae 1–3 pairs per leaf, 4.6–5.1 cm long, with 18–25 pairs of leaflets; leaflets 3.3–13.5 × 1–1.75 mm, linear to linear-oblong, apex sub-acute and mucronate, base oblique, lamina glabrous. **Inflorescence**: an axillary spike, 3.5–5.5 cm long, solitary or in fascicles on short shoots or occupying terminal portions of shoots and produced when the plant is leafless. **Flowers**: dark purple, sessile to sub-sessile; calyx 2.5 mm long, deeply toothed, glabrous; petals 3.5–6 mm long; stamen filaments 6–8 mm long. **Fruit**: a torulose, laterally compressed, falcate craspedium, 25–28 × 3–3.4 cm, with transverse septa between seeds dividing the fruit into one-seeded segments which, upon ripening, fall from the persistent replum. **Seeds**: 10 × 6.5 mm, with pleurogram.

#### Distribution.

Zambia, Tanzania.

#### Habitat and ecology.

Rocky hillsides, especially those of the escarpment facing Lake Tanganyika, in deciduous thicket, scrub and dry evergreen woodland, occasionally on sandy soil. Leafless when flowering.

### ﻿ Entada obliqua

Taxon classificationPlantaeFabalesFabaceae

(Burtt Davy) S.A. O’Donnell & G.P. Lewis comb. nov.

38276431-338C-5AB7-8D07-B9994708ACBC

urn:lsid:ipni.org:names:77303570-1

= Elephantorrhiza obliqua Burtt Davy var. glabra E. Phillips, Bothalia 1: 189. 1923.

#### Type.

SOUTH AFRICA. Transvaal, between Carolina and Oshoek, ~ 1.6 km from Robinson’s Farm, *J. Burtt Davy 2976* (holotype: BM [BM000081856]; isotypes: FHO, K [K000232281]).

#### Basionym.

*Elephantorrhizaobliqua* Burtt Davy, Bull. Misc. Inform. Kew 1921: 191. 1921.

#### Description.

Geoxylic suffrutex with erect, annual, usually unbranched stems up to 30 cm from underground axes, stems pubescent to glabrous. **Leaves**: primary and secondary axes glabrous to sparsely pubescent; petiole 2–6 cm long; rachis (0–)1.5–9 cm long; pinnae (1–)2–6 pairs per leaf, 2–11 cm long, with 4–13(–21) pairs of leaflets; leaflets 5.5–15 × 2–6.5 mm, distinctly asymmetric, ovate to oblong-ovate, apex acute or mucronate, base oblique, mid-rib running from distal corner of leaflet base to apex centre, lamina glabrous. **Inflorescence**: an axillary spiciform raceme, 3.5–6 cm long, solitary, rachis glabrous to sparsely pubescent. **Flowers**: yellowish-white, pedicels 1.5 mm long, with minute red glands at base; calyx campanulate, 2 mm long, shallowly toothed, glabrous; petals 4.5 mm long; stamen filaments 7.5 mm long. **Fruit**: a laterally compressed, straight craspedium, 11 × 4 cm, lacking transverse septa between seeds, thus leaving the valves to separate from the replum intact upon ripening, the epicarp exfoliating from the endocarp. **Seeds**: mature seeds not seen.

#### Distribution.

South Africa, restricted to the Transvaal.

#### Habitat and ecology.

In grassland.

### ﻿ Entada parvifolia

Taxon classificationPlantaeFabalesFabaceae

Merr., Philipp. J. Sci., C 3: 229. 1908.

D3553D70-48DA-553D-AD42-AB40ED53497C

= Entada philippinensis Gagnep., Notul Syst. (Paris) 2: 58. 1911.

#### Type.

PHILIPPINES. Luzon, Zambales Province, *M. Ramos 5067* (holotype: NY [NY00002028]; isotypes: K [K000295958], US [US01108049]).

#### Description.

Shrub, scandent, stem swollen from base, tuberous. **Leaves**: rachis 4–7.5 cm long; pinnae 2 pairs per leaf, 4.5–7.5 cm long, with 8–11 pairs of opposite leaflets; leaflets 1.1–1.9 × 0.4–0.75 cm, obliquely oblong, asymmetric, apex rounded to truncate, retuse or mucronate, base cuneate to rounded, lamina glabrous above and below. **Inflorescence**: a supra-axillary, 15 cm long spike, axis appressed-puberulous. **Flowers**: sub-sessile, staminate or bisexual; calyx cupular, 1 mm long, with minutely deltate teeth, glabrous to sparsely puberulent; petals 3 mm long, oblong; stamen filaments 5.5–7 mm long. **Fruit**: a straight, torulose craspedium, 29.5 × 5–5.5 cm, with transverse septa between seeds dividing the fruit into one-seeded segments which, upon ripening, fall from the persistent replum; epicarp chartaceous, endocarp papyraceous. **Seeds**: irregularly ovoid, 1.8 × 1.6 × 0.8 cm, dark brown, lacking a pleurogram.

#### Distribution.

Philippines.

#### Habitat and ecology.

Low elevation thickets.

### ﻿ Entada pervillei

Taxon classificationPlantaeFabalesFabaceae

(Vatke) R. Vig., Notul. Syst. (Paris) 13: 347. 1949, pro parte, var. louvellii excl. (see E. louvellii)

266092AE-35D1-5F7A-8603-6024108C9ACC

≡ Entada pervillei var. genuina R. Vig., Notul. Syst. (Paris) 13: 347. 1949. Nom. superfl.

#### Type.

MADAGASCAR. Nossi Bé [Nosy Bé], *J.M. Hildebrandt 2952* (holotype: B?; isotypes: JE [JE00003317, JE00003318], K, M [M0218736], P).

#### Basionym.

Piptadenia?pervillei Vatke, Linnea 43: 109. 1881.

#### Description.

Tree to 15 m tall, with elevated nectaries at nodes. **Leaves**: rachis 8–18 cm long, ridged above, sometimes with elevated nectaries between distal pairs of pinnae, tendrils lacking; pinnae 7–16 pairs per leaf, 3.5–11 cm long, with 26–72 pairs of leaflets; leaflets (4–)6–10.5 × 1–1.5 mm, linear-oblong, sub-falcate, apex acute to rounded or obtuse, base asymmetric, rounded on the proximal margin, attenuate on the distal margin, lamina glabrous, margins ciliolate to ciliate at base. **Inflorescence**: a terminal panicle of spikes, each spike 7–25 cm long, spike rachis slightly pubescent. **Flowers**: white, sub-sessile; calyx obconical, 1–1.6 mm long, shallowly toothed, glabrous; petals 2.5–4 × 1 mm; stamen filaments 5–6.5 mm long. **Fruit**: a torulose, laterally compressed craspedium, 18–25 × 2.5–4.5 cm, with transverse septa between seeds dividing the fruit into one-seeded segments which, upon ripening, fall from the persistent replum. **Seeds**: oblong-ovate, 1.7 × 1 cm, brown, pleurogram indistinct.

#### Distribution.

Madagascar (north, northeast and west).

#### Habitat and ecology.

Humid evergreen forest and seasonally dry deciduous woodland up to 700 m alt.; sandy or calcareous soils.

#### Note.

The “?” in the basionym Piptadenia?pervillei Vatke is associated with the genus *Piptadenia* and not with the species name *pervillei* because Vatke was not certain about the generic position of the species. Entadapervilleivar.genuina R. Vig. (i.e. equivalent to the typical variety var. pervillei ) is a superfluous name because, once var. louvellii was moved to *E.louvellii*, the typical variety was effectively disbanded.

### ﻿ Entada phaneroneura

Taxon classificationPlantaeFabalesFabaceae

Brenan, Kew Bull. 32: 545. 1978.

F7D8D18D-FC42-5458-8130-B7183CB92229

#### Type.

BURUNDI. Bubanza Territory, Cibitoke, *J. Lewalle 3238* (holotype: K; isotypes: BR [BR0000008915856], FHO).

#### Description.

Shrub, climbing to 12 m. **Leaves**: rachis (2–)4–5 cm long, glabrous, terminating in a bifurcating tendril; pinnae 2 pairs per leaf, (1.5–)3–4 cm long, with 9–15 pairs of leaflets, pinna rachis distinctly winged; leaflets 5–8(–16) × 1.5–4 mm, oblong-oblanceolate to near linear, apex rounded to obtuse and mucronate, base oblique, lamina glabrous. **Inflorescence**: an axillary spiciform raceme, 5–6 cm long, the racemes often aggregated into a panicle, rachis glabrous. **Flowers**: purple, pedicels 1–2 mm long; calyx 0.75–1 mm long, distinctly toothed, glabrous; petals 3 × 1.1–1.2 mm; stamen filaments 4–5 mm long. **Fruit**: a torulose, laterally compressed, falcate craspedium, 20 × 3–5 cm, with transverse septa between seeds dividing the fruit into one-seeded segments which, upon ripening, fall from the persistent replum. **Seeds**: mature seeds not seen (although several specimens in BR have fruits).

#### Distribution.

Burundi, Democratic Republic of Congo.

#### Habitat and ecology.

Wooded savannah, ×erophilous thickets and dry forest; 800–950 m alt.

### ﻿ Entada phaseoloides

Taxon classificationPlantaeFabalesFabaceae

(L.) Merr., Philipp. J. Sci., C 9: 86. 1914.

7CD3FD95-B91A-51CD-89C6-7071181EB75E

= Entada gandu Hoffmanns., Verz. Pfl.–Kult. 8: 274. 1824.
= Entada parrana Spreng., Syst. Veg. 2: 325. 1825.
= Entada adenanthera DC., Mém. Légum.: 422. 1826.
= Entada scandens (L.) Benth., J. Bot. (Hooker) 4: 332. 1841.
= Entada rumphii Scheff., Natuurk. Tijdschr. Ned.–Indië 32: 412. 1871.
= Entada scandens var. aequilatera Domin, Biblioth. Bot. 22(89): 247. 1926.

#### Type.

INDONESIA. Maluku, Amboina, illustration of *Faba marina major* in Rumphius Herb. Amb. 5: 5–8, tab. 4. 1747.

#### Basionym.

*Lensphaseoloides* L., Herb. Amboin. (Linn.) 18. 1754.

#### Description.

**Figure 18.**
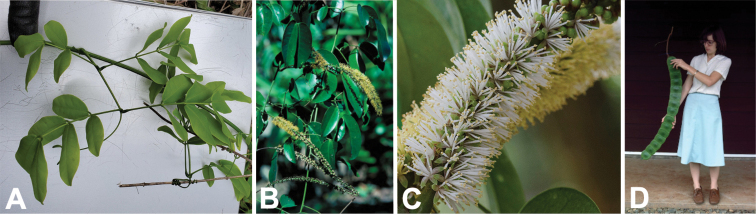
*Entadaphaseoloides* vegetative and reproductive structures. **A** leaf with rachis terminating in a bifurcating tendril, Guam (photo: Pacific Island Network (PACN), US National Park Service (NPS) *CC BY-NC 4.0*, Ueda (2021) observation 34434359) **B** shoot bearing leaves and axillary flower spikes, Australia (photo: photographer unknown, Centre for Australian National Biodiversity Research (CANBR), 1998) **C** open, sessile flowers, Hong Kong (photo: C Chiu, all rights reserved, Chiu (2021)) **D** nearly mature pod, Australia (photo: B Gray, Centre for Australian National Biodiversity Research (CANBR), 1979).

Liana to 40 m long, stems often flattened and spirally twisted, with pit nectaries at nodes. **Leaves**: petiole 1.5–3.5 cm long, rachis 4.3–7.7 cm long, terminating in a bifurcating tendril; pinnae 1–2 pairs per leaf, 6–20 cm long, each pinna with 1–2(–3) pairs of leaflets; leaflets opposite, coriaceous, elliptic or narrowly obovate, sometimes asymmetrical about the mid-vein, 4.5–10 × 1.8–6.3 cm and increasing in size distally, apex acute to acuminate, retuse, base obtuse, mid-rib and margins puberulous (Fig. 18A). **Inflorescence**: a spike, 11.5–30 cm long, axillary, solitary or fascicled on short shoots, puberulous (Fig. 18B). **Flowers**: sessile to sub-sessile, staminate or bisexual, mildly fragrant; calyx cupular, glabrous, 0.8–1.2 mm long; petals green with base reddish; stamen filaments 4–6.5 mm long, white turning yellow; ovary slender, glabrous (Fig. 18C). **Fruit**: a gigantic, torulose craspedium, 100–135(–200) × 7–15 cm, straight to slightly curved, with transverse septa between seeds dividing the fruit into one-seeded segments which, upon ripening, fall from the persistent replum; segments 6.5–7.5 cm long; epicarp woody, endocarp chartaceous; 9–16-seeded (Fig. 18D). **Seeds**: subcircular, laterally compressed, but convex with an angular margin, 3.5–5.5 × 3.3–4.5 × 1–1.5 cm, hard, reddish-brown, pleurogram lacking; an air-filled cavity between the cotyledons.

#### Distribution.

Subtropical Japan (Ryukyu Islands), Taiwan (south), throughout Malesia, Australia (east coast of northern Queensland), Micronesia, southwest Pacific.

#### Habitat and ecology.

A wide variety of habitats from back-mangrove and lowland freshwater swamp, riparian vegetation and lowland rainforest up to montane forest, 0–1700 m alt.

### ﻿ Entada polyphylla

Taxon classificationPlantaeFabalesFabaceae

Benth., J. Bot. (Hooker) 2: 133. 1840.

B00A0463-BF81-50BF-94F7-B3D3CAF83182

= Entada paranaguana Barb. Rodr., Vellosia, ed. 2, 1: 18. 1891.
= Entada polystachya var. polyphylla (Benth.) Barneby, Brittonia 48: 175. 1996.

#### Type.

GUYANA. Rio Quitaro, *R.H. Schomburgk 604* (holotype: K [K000504673, K000504674]; isotypes: E [E00296969], F [F0092593F], NY [NY00002025], US [US00001028]).

#### Description.

Shrub, scandent, to 10 m. **Leaves**: rachis 7–13 cm long, puberulous, tendrils lacking; pinnae 4–7 pairs per leaf, 5–7 cm long, with (12–)13–20 pairs of leaflets; leaflets 8–20 × 3–8 mm, oblong, apex rounded to emarginate, base truncate to subtruncate, lamina pubescent above and below (Fig. 2G). **Inflorescence**: a compound, terminal, one-sided panicle of up-turned spikes, each spike 4–6.5 cm long, rachis pubescent (Fig. 2G). **Flowers**: cream to greenish-yellow, staminate or bisexual, sub-sessile; calyx cupular, 0.5–1 mm long, glabrous to sparsely puberulous; petals 2.5–3 × 0.8–1 mm; stamen filaments 3–4 mm long. **Fruit**: a torulose, laterally compressed craspedium, 20–30 × 6 cm, with transverse septa between seeds dividing the fruit into one-seeded segments which, upon ripening, fall from the persistent replum. **Seeds**: 1.9–2.4 × 1–1.2 cm, with pleurogram.

#### Distribution.

Amazonian Brazil, Ecuador, Peru, Venezuela, the Guianas, Puerto Rico.

#### Habitat and ecology.

Disturbed forest, grassy fields, secondary vegetation at forest margins.

### ﻿ Entada polystachya

Taxon classificationPlantaeFabalesFabaceae

(L.) DC., Mém. Légum. 434. t. 61. 1825.

260BA5F0-5CC0-5A66-93D8-63CD2A034AD0

= Entada chiliantha DC., Mém. Légum. 422. 1826.
= Entada plumeri Spreng., Syst. Veg. 4(2): 164. 1827.
= Entada acaciifolia Benth., Trans. Linn. Soc. London 30: 365. 1875.

#### Type.

illustration in Plumier, Pl. Amer. 1: tab. 12. 1755.

#### Basionym.

*Mimosapolystachya* L., Sp. Pl. 1: 520. 1753.

#### Description.

**Figure 19.**
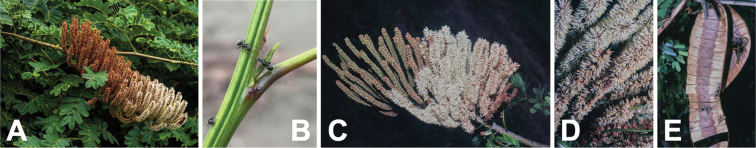
*Entadapolystachya* vegetative and reproductive structures. **A** branch and shoots of scandent shrub bearing leaves and a terminal one-sided panicle of up-turned flower spikes, Ecuador (photo: M Alache, all rights reserved, iNaturalist (2021) observation 95175614) **B** stem node with ants accessing gland at point of petiole insertion, Costa Rica (photo: J Montero, all rights reserved, iNaturalist (2021) observation 86105886) **C** terminal panicle of up-turned flower spikes, with open flowers appearing white and buds appearing brown, Brazil (photo: G Lewis) **D** open, sessile flowers, Brazil (photo: G Lewis) **E** mature pods, Brazil (photo: G Lewis).

Liana or scandent shrub to 10 m. **Leaves**: rachis 6–13 cm long, glabrous to puberulous; pinnae (2–)3–5 pairs per leaf, 3.5–8 cm long, with 5–11 pairs of leaflets; leaflets 1.5–4 × 0.5–2 cm, oblong, apex rounded, base oblique, lamina glabrous above and below (Fig. 19A). **Inflorescence**: a terminal one-sided panicle of up-turned spikes, each spike 8–10 cm long, spike rachis glabrous to puberulous (Fig. 19A, C). **Flowers**: cream-coloured (the stamens) and reddish (the sepals and petals), with an unpleasant odour; calyx cupular, 1 mm long; petals 2.5–4 × 0.8–1 mm; stamen filaments 4 mm long (Fig. 19D). **Fruit**: a torulose, laterally compressed, falcate craspedium 15–30(–40) × (5–)5.5–9.3 cm, with transverse septa between seeds dividing the fruit into one-seeded segments which, upon ripening, fall from the persistent replum; mesocarp over seeds conspicuous and spongy (Fig. 19E). **Seeds**: elliptic, 1.2–1.7 × 0.8–1.3 × 2–4 mm, with pleurogram.

#### Distribution.

Pacific Mexico east to Lesser Antilles and south to Bolivia.

#### Habitat and ecology.

Seasonally dry and humid forest near the coast, especially on the margins of mangroves, occasionally reaching the forest canopy.

### ﻿ Entada praetermissa

Taxon classificationPlantaeFabalesFabaceae

(J.H. Ross) S.A. O’Donnell & G.P. Lewis comb. nov.

B85F8CF0-C6D2-50BD-801E-3FC6CF8B6DFC

urn:lsid:ipni.org:names:77303571-1

#### Type.

SOUTH AFRICA. Transvaal, Lydenburg District, Steelpoort Valley, near Sarahshof, *L.E.W. Codd 9830* (holotype: PRE [PRE0391104-0]; isotypes: BM [BM000842179], K [K000232268]).

#### Basionym.

*Elephantorrhizapraetermissa* J.H. Ross, Bothalia 11: 252. 1974.

#### Description.

Shrub 1–2 m tall. **Leaves**: petiole 2.2– 4 cm long; rachis 4–9 cm long, grooved above and with occasional scattered dark glands; pinnae (3–)5–10(–12) pairs per leaf, (2.8–)3.5–6(7) cm long, with 20–40 pairs of leaflets; leaflets 5–10 × 0.9–1.5 mm, linear to linear-oblong, apex rounded to acute, base oblique, mid-rib running from distal corner of leaflet base to apex centre, lamina glabrous. **Inflorescence**: a spiciform raceme, 4–5.5 cm long, solitary or aggregated in fascicles or on short lateral shoots, rachis glabrous. **Flowers**: yellowish-white; pedicels 1.5–2 mm long, articulated near or below the middle, with minute reddish glands at the base; calyx 0.75–1.25 mm long, toothed, glabrous; petals 2–3 mm long; stamen filaments 4–5 mm long. **Fruit**: a laterally compressed, straight to slightly curved craspedium, 12–18 × 2–3.2 cm, lacking transverse septa between seeds, thus leaving the valves to separate from the replum intact upon ripening, the epicarp exfoliating from the endocarp. **Seeds**: laterally compressed, 15 × 13 × 3.5 mm.

#### Distribution.

South Africa, apparently restricted to the Transvaal.

#### Habitat and ecology.

On dry wooded hillsides.

### ﻿ Entada rangei

Taxon classificationPlantaeFabalesFabaceae

(Harms) S.A. O’Donnell & G.P. Lewis comb. nov.

B084EF3E-C265-5B27-902C-9242EAD98CC7

urn:lsid:ipni.org:names:77303572-1

= Elephantorrhiza suffruticosa Schinz, Mém. Herb. Boissier 8: 117. 1900, non EntadasuffruticosaVatke. 1881 [= Mimosasuffruticosa (Vatke) Drake]. Type: ANGOLA. Huila District, “Kilevi am Kunene”, south of Humbe, *Schinz 2071* (lectotype: Z, designated by J.H. Ross in Fl. Southern Afr. 16(1): 148. 1975).

#### Type.

NAMIBIA. Keetmanshoop District, Naute, near Keetmanshoop, *P. Range 455* (holotype: B†; drawing: BM [BM000842180]; isotypes: BOL, NBG [SAM0073417-1, SAM0073417-2], SAM).

#### Basionym.

*Elephantorrhizarangei* Harms, Bot. Jahrb. Syst. 49(3–4): 420. 1913.

#### Description.

**Figure 20.**
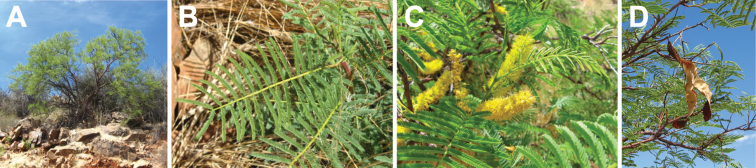
*Entadarangei* habit, vegetative and reproductive structures. **A** erect shrub, Namibia (photo: A Dreyer, Dressler et al. (2014a)) **B** short shoot and adaxial view of mature leaves, Namibia (photo: A Dreyer, Dressler et al. (2014a)) **C** spiciform racemes of open flowers amongst young leaves, Namibia (photo: A Dreyer, Dressler et al. (2014a)) **D** mature pods, Namibia (photo: A Dreyer, Dressler et al. (2014a)).

Shrub or small tree, 1–6 m tall (Fig. 20A). **Leaves**: petiole (0.6–)1.5–3.5 cm long; rachis (0.5–)10–17(–25.4) cm; pinnae (2–)15–27(–42) pairs per leaf, (1.4–)2–3.5(–6.8) cm long, with (17–)27–40(–50) pairs of leaflets; leaflets 3–7.5 × 0.4–1.2 mm, linear-oblong to linear, apex obtuse to acute, asymmetric and often mucronate, base oblique with proximal margin rounded, mid-rib marginal throughout or more rarely running from the distal corner of the leaflet base to the apex centre, lamina glabrous (Fig. 20B, C). **Inflorescence**: an axillary spiciform raceme, (4–)6–14(–18) cm long, 1–3 per axil or borne on short lateral shoots, rachis pubescent or sometimes glabrous (Fig. 20C). **Flowers**: yellowish-white, golden yellow or cream-coloured; pedicels 1 mm long, articulated near the middle, with minute reddish, reddish-brown or pale yellow glands at the base of the pedicels; calyx cupular, 1 mm long, shallowly toothed, glabrous; petals 3–3.75 mm long; stamen filaments 5 mm long. **Fruit**: a laterally compressed, straight to slightly curved craspedium, 8.5–30.5 × 1.8–2.25 cm, transverse veins usually prominent, lacking transverse septa between seeds, thus leaving the valves to separate from the replum intact upon ripening, the epicarp of both valves peeling away from the endocarp; umbonate over seeds (Fig. 20D). **Seeds**: ellipsoid, 13–15 × 9–12 mm.

#### Distribution.

Angola, Namibia, Zimbabwe, Mozambique.

#### Habitat and ecology.

Woodland and grassland, often in rocky areas; 1050–2130 m alt.

#### Note.

*Elephantorrhizarangei* Harms was treated as a distinct species by Ross (1975a), although with some hesitation because the species was known only from the type locality and displays considerable variation in leaflet size, even on a single branch. Ross also noted that it “bears a superficial resemblance to *Elephantorrhizasuffruticosa* Schinz.” Plants of the World Online (POWO) places *Elephantorrhizarangei* as a synonym of *Elephantorrhizasuffruticosa* Schinz, but when transferred to the genus *Entada*, the epithet *suffruticosa* cannot be used because the name *Entadasuffruticosa*Vatke (1881, p. 108), for a Madagascan species (a synonym of *Mimosasuffruticosa* (Vatke) Drake), already exists.

### ﻿ Entada reticulata

Taxon classificationPlantaeFabalesFabaceae

Gagnep., Notul. Syst. (Paris) 2: 59. 1911.

3A48D39A-EE1E-5EEE-B8A6-3CBE38433A21

#### Type.

LAOS. Bassac, *Thorel 1427*, *p.p*. (holotype: P [P030131, P030132]).

#### Description.

Shrub, scandent. **Leaves**: petiole 1.3–2.3 cm long, rachis 3.5–5 cm long, terminating in a bifurcating tendril; pinnae 2 pairs per leaf, 5–7 cm long, with 8–16 pairs of opposite leaflets; leaflets 0.6–1.8 × 0.2–0.4 cm, oblong, apex mucronate, base obtuse, lamina glabrous except for pubescence on mid-rib below. **Inflorescence**: a 5–8 cm long, axillary, solitary spike, axis pubescent. **Flowers**: sessile, staminate or bisexual; calyx cupular, 0.8–1.5 mm long, shallowly toothed, glabrous; petals 3–3.5 mm long, linear-lanceolate. **Fruit**: a straight to slightly curved torulose craspedium, 5.5–11.5 × 1.5 cm, with transverse septa between seeds dividing the fruit into one-seeded segments which, upon ripening, fall from the persistent replum; segments 1.3–1.5 cm long; epicarp coriaceous, endocarp parchment-like. **Seeds**: globose, 0.85 cm in diameter, hard, brown, pleurogram lacking.

#### Distribution.

Laos, Cambodia.

#### Habitat and ecology.

Seasonally dry deciduous forest or mixed forest with Dipterocarpaceae.

### ﻿ Entada rheedei

Taxon classificationPlantaeFabalesFabaceae

Spreng., Syst. Veg. 2: 325. 1825.

8DCC3817-207E-5061-AE4E-FBCDFE7EBB54

= Entada pursaetha DC., Mém. Légum.: 421. 1826.
= Entada monostachya DC., Mém. Légum.: 422. 1826.
= Entada gogo I.M. Johnst., Sargentia 8: 137. 1949.

#### Type.

INDIA. Malabar coast, illustration in *Rheede* Hort. Malab. 9: 151, tab. 77. 1689.

#### Description.

**Figure 21.**
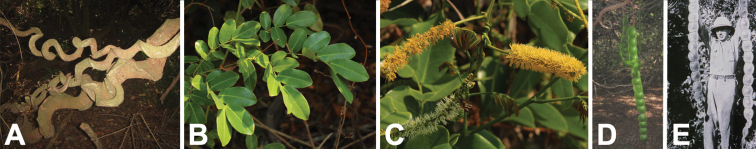
*Entadarheedei* habit, vegetative and reproductive structures. **A** liana with twisted woody stems, South Africa (photo: R Taylor *CC BY-NC 4.0*, Ueda (2021) observation 28499551) **B** adaxial view of mature leaf, South Africa (photo: R Taylor *CC BY-NC 4.0*, Ueda (2021) observation 11095941) **C** axillary spikes of open, sessile flowers, South Africa (photo: R Taylor *CC BY-NC 4.0*, Ueda (2021) observation 11095941) **D** immature pods, South Africa (photo: R Taylor *CC BY-NC 4.0*, Ueda (2021) observation 28499551) **E** mature pods of collection Schleiben 769a, Tanzania (photographer unknown).

Liana to 75 m long, stems to 30 cm diameter at base (Figs 2A, 21A), with elevated nectaries at nodes. **Leaves**: rachis 6.4–12.9 cm long, terminating in a bifurcating tendril; pinnae 1–2 pairs per leaf, 5–14 cm long, with 3–5 pairs of opposite leaflets, sometimes terminating in a glandular mucro (Fig. 4E); leaflets 1.8–6.6 × 1.2–2.9 cm, chartaceous, elliptic to oblanceolate, asymmetric, apex obtuse to acuminate, retuse, base rounded to attenuate, mid-rib above pubescent, lamina glabrous, except below near the base (Figs 2E, 21B). **Inflorescence**: a spike, 8–25 cm long, axillary, solitary, or sometimes several spikes from a short shoot, peduncle 1–8.5 cm long, peduncle and rachis puberulous to villose (Fig. 21C). **Flowers**: cream or greenish, sessile to sub-sessile, staminate or bisexual, with an unpleasant odour; calyx cupular, 0.75–1.2 mm long, shallowly toothed; petals pale green to white, 2.5–3.5 mm long; stamen filaments 2–6.5 mm long, white turning yellow; stigma shallowly cupular. **Fruit**: a gigantic, torulose craspedium, 50–200 × 7–15 cm, straight to slightly curved, with transverse septa between seeds dividing the fruit into one-seeded segments which, upon ripening, fall from the persistent replum; segments 6.5–7.5 cm long; epicarp and endocarp woody (Figs 2J, 21D, E). **Seeds**: subcircular, laterally compressed, 5 × 3.5–5 cm, hard, brown, pleurogram lacking.

### ﻿ subsp. rheedei

Taxon classificationPlantaeFabalesFabaceae

CC881637-8AD0-5EE5-8043-F79466E0A92D

#### Description.

Calyx glabrous.

#### Distribution.

Tropical and southern subtropical Africa (including Madagascar), Mascarene Islands, Sri Lanka, India, Bangladesh, mainland South East Asia, southern China, Taiwan, Malesia, tropical northern Australia.

#### Habitat and ecology.

Primary and secondary rainforest, especially riparian, back-mangrove and beach forest, 0–900 m alt.

### ﻿ subsp. sinohimalensis

Taxon classificationPlantaeFabalesFabaceae

(Grierson & D.G. Long) S.A. O’Donnell & G.P. Lewis comb. nov.

9DFD21CB-2FB5-582E-BFCF-B453A2552441

urn:lsid:ipni.org:names:77303573-1

≡ Entada pursaetha var. sinohimalensis (Grierson & D.G. Long) C. Chen & H. Sun, Fl. Yunnanica 10: 289. 2006.
= Entada laotica Gagnep., Bull. Soc. Bot. France 99: 46. 1952.

#### Type.

NEPAL. Without locality, *N. Wallich 5294a* (holotype: K [K000756992]; isotypes: BM, E).

#### Basionym.

Entadapursaethasubsp.sinohimalensis Grierson & D.G. Long, Notes Roy. Bot. Gard. Edinburgh 37: 348. 1979.

#### Description.

Calyx puberulous to velutinous.

#### Distribution.

Nepal, northeast India, Bangladesh, Myanmar, Laos, southwest China (Yunnan).

#### Habitat and ecology.

Wet forest, especially riparian, up to about 1300 m alt.

### ﻿ Entada schinziana

Taxon classificationPlantaeFabalesFabaceae

(Dinter) S.A. O’Donnell & G.P. Lewis comb. nov.

DE86F9C6-8B61-50F2-A85D-04B27A7004AC

urn:lsid:ipni.org:names:77303574-1

#### Type.

NAMIBIA. Grootfontein District, Otavi, *Dinter 745* (lectotype: SAM [SAM0073418-0], designated by J.H. Ross in Fl. Southern Afr. 16(1): 148. 1975).

#### Basionym.

*Elephantorrhizaschinziana* Dinter, Repert. Spec. Nov. Regni Veg. 17: 190. 1921.

#### Description.

Branched shrub to 2.5 m tall. **Leaves**: petiole 2.2–3.5(–5.2) cm long; rachis (4.5–)7.5–14.5(–20.5) cm long; pinnae (2–)6–11(–14) pairs per leaf, 5.5–10(–14) cm long, with (14–)21–40 pairs of leaflets; leaflets (5–)7–14 × 1.5–3.5 mm, linear-oblong to oblong, apex rounded and sometimes mucronate, base oblique, mid-rib running from distal corner of leaflet base to apex centre, lamina glabrous, slightly glaucous. **Inflorescence**: an axillary spiciform raceme, 7–9.5 cm long, 1–2 per axil, rachis glabrous. **Flowers**: yellowish-white; pedicels 0.75 mm long, articulated towards the apex, with minute yellowish glands at the base; calyx cupular, 1.5 mm long, shallowly toothed, glabrous; petals 3–3.75 mm long; stamen filaments 5 mm long. **Fruit**: a laterally compressed, straight to slightly curved craspedium, (15–)19–30(–40.5) × 3–3.9 cm, transverse veins prominent, lacking transverse septa between seeds, thus leaving the valves to separate from the replum intact upon ripening, the epicarp of both valves peeling away from the endocarp; umbonate over seeds. **Seeds**: mature seeds not seen.

#### Distribution.

Namibia.

#### Habitat and ecology.

In savannah and woodlands.

#### Note.

Ross (1975a, p. 148) noted that the above description of the flowers of *E.schinziana* comes from the second sheet of *Dinter 1689*, which Ross regarded as of potentially ambiguous identity given that “one of the [other two] sheets of *Dinter 1689* is a mixed gathering of a vegetative shoot of *E.suffruticosa* and a pod of *E.schinziana*”. The flowering specimen on the second sheet is leafless, thus preventing a more definitive identification. Ross conceded that “it is possible therefore that the flowers described are those of *E.suffruticosa* and not of *E.schinziana*.”

### ﻿ Entada simplicata

Taxon classificationPlantaeFabalesFabaceae

(Barneby) Sch. Rodr. & A.S. Flores, Phytotaxa 39: 47. 2012.

68ACBB0D-DDE9-5B12-A3E7-6ECD19E78F60

#### Type.

BRAZIL. Roraima, Municipality Caracaraí, North Perimetral Road (BR–210) 10 km from the junction with the Manaus–Caracaraí Road (BR–174), near Novo Paraiso, *C.A. Cid Ferreira 9220* (holotype: INPA; isotype: NY [NY00038703]).

#### Basionym.

Entadapolystachyavar.simplicata Barneby, Brittonia 48: 175. 1996.

#### Description.

Liana or scandent shrub to 10 m. **Leaves**: petiole 3.7–7.7 cm long, rachis 5.8–13 cm long; pinnae 1–3 pairs per leaf, 1.6–3.7 cm long, with 1–3 pairs of leaflets; leaflets 2.5–8.3 × (1.8–)2.2–5.2 cm, obovate to broadly elliptic, apex retuse to truncate, base asymmetric, rounded to cuneate, both surfaces glabrous. **Inflorescence**: a terminal one-sided panicle of up-turned spikes, each spike rachis 16–26 cm long. **Flowers**: calyx 1–1.2 mm long, shallowly toothed; petals 2.2–3.3 mm long; stamen filaments 3.8–4.5 mm long. **Fruit**: a torulose, laterally compressed craspedium, 25.5–29 × 3.8–5.3 cm, with transverse septa between seeds dividing the fruit into one-seeded segments which, upon ripening, fall from the persistent replum. **Seeds**: elliptic, 14–20 × 9–13 mm, with pleurogram.

#### Distribution.

Brazil (Roraima State).

#### Habitat and ecology.

Open margins of wet tropical forest on rocky slopes.

### ﻿ Entada spinescens

Taxon classificationPlantaeFabalesFabaceae

Brenan, Kew Bull. 10: 168. 1955.

7C8B28AC-84E2-57DE-9ECF-751F724B08A0

#### Type.

TANZANIA. Mpwapwa District, near Gulwe, *B.D. Burtt 4639* (holotype: K [K000232157, K000232158]).

#### Description.

Climber, slender, woody to 3.6 m, stipules spinescent, young shoots pubescent. **Leaves**: stipules sub-conical, spinescent, rigid, gradually spreading, rachis 3.4–10.7 cm long; pinnae 1–3 pairs per leaf, sometimes modified into a tendril or spirally twisted at base, each pinna 2.8–6 cm long, with 12–18 pairs of leaflets; leaflets 5.6–17.5 × 1.7–3.2 mm, oblong to linear-oblong, apex rounded to obtuse and mucronate, base oblique, lamina glabrous, except for puberulous mid-rib and margins. **Inflorescence**: an axillary spike, 3–7 cm long, solitary, the rachis pubescent. **Flowers**: purple, sub-sessile; calyx 1 mm long, distinctly toothed, glabrous; petals 3–4 × 1.2–1.6 mm; stamen filaments 3.5–4.6 mm long. **Fruit**: a torulose, laterally compressed, falcate craspedium, 13–17 cm long, with transverse septa between seeds dividing the fruit into one-seeded segments which, upon ripening, fall from the persistent replum. **Seeds**: sub-circular to ovate, 10.4 × 9.2 × 2.3 mm, with closed pleurogram.

#### Distribution.

Tanzania.

#### Habitat and ecology.

Deciduous bushland and tall deciduous thickets; 910–1220 m alt.

### ﻿ Entada spiralis

Taxon classificationPlantaeFabalesFabaceae

Ridl., J. Asiat. Soc. Bengal, Pt. 2, Nat. Hist. 67: 305. 1898.

AD0825F1-1F11-5393-A010-602F8F067CFA

#### Type.

Not specified, though Ridley’s description appears to be based upon plants that are “very common in Singapore…[and] very conspicuous here from its very remarkable fruit.” (Ridley 1898, p. 305).

#### Description.

**Figure 22.**
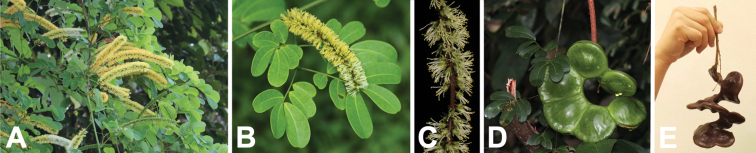
*Entadaspiralis* vegetative and reproductive structures. **A** climbing shoot bearing leaves and flower spikes, Singapore (photo: C Ng *CC BY-NC-SA 2.0*, Ng (2021)) **B** leaf and axillary flower spike, Singapore (photo: C Ng *CC BY-NC-SA 2.0*, Ng (2021)) **C** spike with open, subsessile flowers, Singapore (photo: C Ng *CC BY-NC-SA 2.0*, Ng (2021)) **D** immature, spirally coiled pod, Singapore (photo: C Ng *CC BY-NC-SA 2.0*, Ng (2021)) **E** mature, spirally coiled pod, Singapore (photo: B-C Ho).

Liana more than 25 m long, stem flattened and spirally twisted, 7.5 cm wide × 2.5–5 cm thick. **Leaves**: rachis 5–9 cm long, tomentose, terminating in a bifurcating tendril; pinnae 2–3 pairs per leaf, 3.6–9.3 cm long with 2–4 pairs of opposite leaflets; leaflets 1.8–6.5 × 0.9–3 cm, obovate to narrowly obovate-elliptic, unequal-sided, apex rounded-truncate, retuse, base rounded to cuneate, asymmetrical, lamina chartaceous, glabrous (Fig. 22A, B). **Inflorescence**: a spike 15–20 cm long, axillary, solitary, tomentose (Fig. 22A, B). **Flowers**: sessile to sub-sessile, staminate or bisexual; calyx cupular, 0.5–1 mm long, glabrous to puberulous; petals white, 2.5–3 mm long; stamen filaments 5–8 mm long, white turning yellow (Fig. 22C). **Fruit**: a large, torulose, spirally coiled craspedium, 120–180 × 6 cm, with transverse septa between seeds dividing the fruit into one-seeded segments which, upon ripening, fall from the persistent replum; segments irregularly triangular; epicarp woody, endocarp chartaceous (Fig. 22D, E). **Seeds**: irregularly compressed and mirroring the fruit segment shape, 6–6.5 × 5 cm × 1.5–1.8 cm, hard, brown, pleurogram lacking.

#### Distribution.

Peninsular Thailand, peninsular Malaysia, Singapore, Sumatra.

#### Habitat and ecology.

Primary and secondary rainforest; 0–540 m alt.

### ﻿ Entada stuhlmannii

Taxon classificationPlantaeFabalesFabaceae

(Taub.) Harms, Veg. Erde 9(III 1): 401. 1915.

D5811211-0C16-5993-AF4F-3AA3497B3390

#### Types.

TANZANIA. Uzaramo District, *Stuhlmann 6845*, *6939*, *6965*, *7114* (syntypes: B†); Bagamoyo District, *Stuhlmann 7197* (syntype: B†).

#### Basionym.

*Pusaethastuhlmannii* Taub., Pflanzenw. Ost–Afrikas, C: 196. 1895.

#### Description.

Climber, slender, woody, to 2.5 m, young shoots glabrous and sinuous, roots tuberous. **Leaves**: rachis 5–6.2 cm long; pinnae 2(–3) pairs per leaf, sometimes modified into a tendril or spirally twisted at base, 1.9–3.6 cm long, with 4–5(–8) pairs of leaflets, pinna rachis slightly winged; leaflets 0.9–3 × 0.25–1.5 cm, obovate to oblanceolate-oblong, occasionally narrowly oblong, apex rounded to sub-truncate and with or without a mucro, base oblique, lamina glabrous, lateral venation raised below. **Inflorescence**: an axillary spiciform raceme, (2–)3.5–8 cm long, usually solitary though sometimes in fascicles, rachis glabrous. **Flowers**: purple or brownish-red, pedicels 1–1.5 mm long; calyx 1 mm long, distinctly toothed, glabrous; petals 2.5–4 mm long; stamen filaments 3–3.5 mm long. **Fruit**: a torulose, laterally compressed, falcate craspedium, 12–24(–30) × 2.7–4.3 cm, with transverse septa between seeds dividing the fruit into one-seeded segments which, upon ripening, fall from the persistent replum. **Seeds**: 1 × 0.9 cm.

#### Distribution.

Tanzania, Mozambique.

#### Habitat and ecology.

Scrub around Lake Tanganyika, deciduous bushland, wooded grassland and woodland; 15–1600 m alt.

### ﻿ Entada tonkinensis

Taxon classificationPlantaeFabalesFabaceae

Gagnep., Notul. Syst. (Paris) 2: 60. 1911.

948027A5-D51F-584A-8CE0-64E21F8D2668

≡ Entada phaseoloides subsp. tonkinensis (Gagnep.) H. Ohashi, Taiwania 55: 50. 2010.

#### Type.

VIETNAM. Banton Valley, near Tu-vu, *B. Balansa 2130* (holotype: P [P02436139, P02436140]).

#### Description.

Robust liana, stems often flattened and spirally twisted, base up to 60 cm in diameter. **Leaves**: petiole 1.5–4 cm long, rachis 3–6.5 cm long, terminating in a bifurcating tendril; pinnae (1–)2 pairs per leaf, 10–22 cm long, proximal pinnae with 2 opposite pairs of leaflets, distal pinnae with 2–3 opposite pairs of leaflets, increasing in size distally; leaflets 5–12 × 2.5–6 cm, chartaceous, obliquely elliptic to obovate-elliptic, asymmetrical, apex acute to obtuse. **Inflorescence**: a spike, 9–25 cm long, axillary, solitary or several spikes from a short shoot; peduncle glabrous; rachis puberulous. **Flowers**: sessile to sub-sessile, distylous; short-styled flowers on proximal half of spike, long-styled flowers on distal half of spike; calyx cupular, glabrous, 1.2–2 mm long; petals pale green with a reddish base, 3–3.2 mm long; stamen filaments 5.5–7 mm long, white turning yellow; ovaries of long-styled flowers with 12–18 ovules. **Fruit**: a gigantic torulose craspedium, 50–150 × 9–12 cm, laterally compressed, straight to slightly curved, 9–16-seeded, with transverse septa between seeds dividing the fruit into one-seeded segments which, upon ripening, fall from the persistent replum; segments 6.5–7.5 cm long; endocarp chartaceous. **Seeds**: subcircular, compressed with a rounded margin, 5.2–7.4 × 4.7–5.5 × 1.6–2.3 cm, hard, blackish-purple, pleurogram lacking.

#### Distribution.

Subtropical Japan (Ryukyu Islands), Taiwan (north and central), southern China, northern Vietnam.

#### Habitat and ecology.

Inland evergreen forests, especially riparian, from low to mid-elevations.

### ﻿ Entada tuberosa

Taxon classificationPlantaeFabalesFabaceae

R. Vig., Notul. Syst. (Paris) 13: 346. 1949.

2A368CEC-ABBB-5A77-AC91-D6075DCA66FD

#### Type.

MADAGASCAR. Maevarano, near Majunga (Mahajangal), *H. Perrier de la Bâthie 12906* (lectotype: P [P00367633], designated by J.-F. Villiers in Leguminosae of Madagascar: 2002: 168).

#### Description.

**Figure 23.**
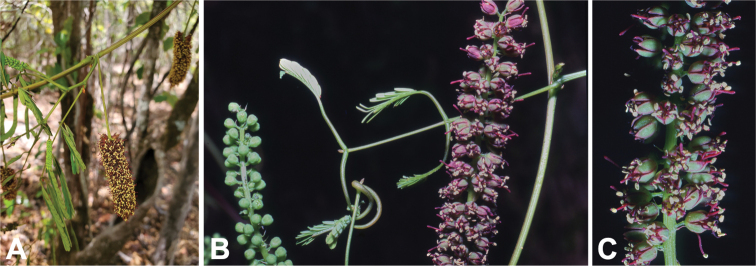
*Entadatuberosa* vegetative and flowering structures. **A** slender climbing shoot bearing leaves and axillary spiciform racemes, Madagascar (photo: feno *CC BY-NC 4.0*, iNaturalist (2021) observation 64636058) **B** spiciform racemes and leaf with twining petiolules, Madagascar (photo: D Du Puy) **C** portion of spiciform raceme with open, pedicellate flowers, Madagascar (photo: B Schrire).

Climber, slender, woody, to 6 m, stem 1 cm in diameter, glabrous or pubescent, twining, with elevated nectaries at nodes; underground tuber elongated. **Leaves**: rachis 5–12.5 cm long, grooved above, laterally winged, glabrous or pubescent, white glandular mucro at apex; pinnae 2–4 pairs per leaf, 2–6.5 cm long, with 13–22 pairs of leaflets; leaflets 5–18 × 1.5–2 mm, oblong, apex rounded to obtuse and mucronate, base oblique, lamina glabrous, mid-rib near distal margin (Fig. 23A, B). **Inflorescence**: a dense, axillary spiciform raceme, 3–7 cm long, solitary or grouped on short leafless shoots or occupying terminal portions of leafy shoots, rachis glabrous or pubescent (Fig. 23A, B). **Flowers**: maroon-red, red-brown or greenish-brown, pedicels 0.75–1.5 mm; calyx obconical, 0.8–1.5 mm long, deeply toothed, glabrous; petals greenish, 3–4.5 mm long; stamen filaments red, 3.5–6.5 mm long (Fig. 23C). **Fruit**: a torulose, laterally compressed, falcate craspedium, 11–23 × 2.9–3.8 cm, 12–14-seeded, with transverse septa between seeds dividing the fruit into one-seeded segments which, upon ripening, fall from the persistent replum. **Seeds**: ovoid, 11 × 9 mm, dark brown, with pleurogram.

### ﻿ var. tuberosa

Taxon classificationPlantaeFabalesFabaceae

42429AC5-29F8-58E3-92C7-B04175AAF0EC

#### Description.

Stem, petiole, leaf rachis, pinna rachis and inflorescence peduncle and rachis glabrous to sparsely pubescent.

#### Distribution.

Madagascar (west, extending to northern tip).

#### Habitat and ecology.

Dry, deciduous woodland and riparian vegetation, on limestone and granite, but not on sand; low altitudes.

### ﻿ var. pubescens

Taxon classificationPlantaeFabalesFabaceae

Brenan, Kew Bull. 20(3): 377. 1966.

AC65D137-B6EC-5378-9DBE-A9C898C8884F

#### Type.

MADAGASCAR. Belambo, near Maeventanana, *H. Perrier de la Bâthie 12129* (holotype: P [P00367634, P00533757]).

#### Description.

Stem, petiole, leaf rachis, pinna rachis and inflorescence peduncle and rachis distinctly to densely pubescent.

#### Distribution.

Madagascar (west).

#### Habitat and ecology.

Dry woodland over granite.

### ﻿ Entada wahlbergii

Taxon classificationPlantaeFabalesFabaceae

Harv., Fl. Cap. 2: 277. 1862.

F2A27EB9-FDFA-55B1-8A26-AE5FB658C9B2

= Entada flexuosa Hutch. & Dalziel, Fl. W. Trop. Afr. 1: 356. 1928.

#### Type.

SOUTH AFRICA. Natal, probably Zululand, *J.A. Wahlberg s.n.* (holotype: S [S13-12053]; photos: K, PRE).

#### Description.

**Figure 24.**
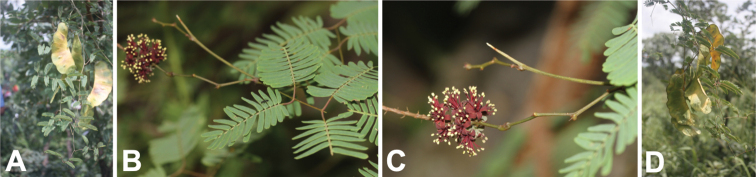
*Entadawahlbergii* vegetative and reproductive structures. **A** slender climbing stem bearing leaves and nearly mature pods, Benin (photo: M Schmidt, Dressler et al. (2014a)) **B** leaf and spiciform raceme, Benin (photo: R Mangelsdorff, Dressler et al. (2014a)) **C** spiciform raceme of open, pedicellate flowers, Benin (photo: R Mangelsdorff, Dressler et al. (2014a)) **D** leaves and nearly mature pods, Benin (photo: M Schmidt, Dressler et al. (2014a)).

Climber, slender, woody, to 3–4 m, young branches glabrous and sinuous (Fig. 24A). **Leaves**: rachis 3.4–8.4 cm long; pinnae (1–)2(–3) pairs per leaf, sometimes modified into a tendril or spirally twisted at base, 2.8–6.5 cm long, with 7–18 pairs of leaflets; leaflets 5–19 × 1.5–6 mm, oblong, apex rounded to obtuse and mucronate, base oblique, lamina glabrous (Fig. 24B). **Inflorescence**: an axillary spiciform raceme, 3–6 cm long, solitary or grouped together on short leafless shoots or occupying terminal portions of leafy shoots, rachis glabrous (Fig. 24C). **Flowers**: dark purple or red, pedicels 1–1.5 mm; calyx 1–1.5 mm long, deeply toothed, glabrous; petals 3–4.5 mm long; stamen filaments 4–6.5 mm long (Figs 2I, 24C). **Fruit**: a torulose, laterally compressed, falcate craspedium, 11–23(–30) × 2.9–3.8(–4.4) cm, with transverse septa between seeds dividing the fruit into one-seeded segments which, upon ripening, fall from the persistent replum (Fig. 24A, D). **Seeds**: 1–1.1 × 0.7–0.8(–1) cm, pleurogram oval, closed.

#### Distribution.

Tropical west to southern Africa, from Guinea and Mali to Nigeria and Sudan, south through the Democratic Republic of Congo, Mozambique and South Africa.

#### Habitat and ecology.

Wooded grassland, open forest, bushveld, valley scrub and banks of dry watercourses on dry, sandy soil; 610–1070 m alt.

### ﻿ Entada woodii

Taxon classificationPlantaeFabalesFabaceae

(E. Phillips) S.A. O’Donnell & G.P. Lewis comb. nov.

63AAA7FF-1EB9-5D96-89DF-8D767D4E6A20

urn:lsid:ipni.org:names:77303575-1

#### Type.

SOUTH AFRICA. Natal, Klip River District, Pieters, near Colenso, *J. Medley-Wood 7958* (holotype: NH [NH0008767-0]; isotype: PRE [PRE0392009-0]).

#### Basionym.

*Elephantorrhizawoodii* E. Phillips, Bothalia 1: 193. 1923.

#### Description.

Geoxylic suffrutex with procumbent, annual, branched, longitudinally striate stems to 60 cm, arising from an elongate subterranean axis, glabrous or pubescent. **Leaves**: petiole 0.8–1.6 cm long, glabrous or pubescent; rachis (1–)3.5–8.5(–13) cm long, grooved above, glabrous or pubescent; pinnae (2–)5–10 pairs per leaf, 1.8–6 cm long, with 12–28 pairs of leaflets; leaflets 2.5–6(–9) × 1–1.8(–2.25) mm, linear to linear-oblong, apex acute to obtuse, sometimes asymmetric, mucronate, base oblique, mid-rib running from distal corner of leaflet base to apex centre, lamina glabrous. **Inflorescence**: an axillary spiciform raceme, 4.5–9.5 cm long, usually solitary, rachis glabrous to densely pubescent. **Flowers**: yellowish-white, pedicels 1.25 mm long and articulated near the middle, with minute glands at the base; calyx 1.5 mm long, shallowly toothed, glabrous; petals 3.25 × 1.25 mm; stamen filaments 6 mm long. **Fruit**: a laterally compressed falcate craspedium, 9 × 3.2 cm, transverse veins prominent, lacking transverse septa between seeds, the valves thus separating from the replum intact upon ripening, the epicarp of both valves peeling away from the endocarp; umbonate over seeds. **Seeds**: mature seeds not seen.

### ﻿ var. woodii

Taxon classificationPlantaeFabalesFabaceae

924D225A-60AC-5AFB-890D-58BABE2EE50E

#### Description.

Stems, petiole, leaf rachis, pinna rachis and inflorescence peduncle and rachis glabrous or almost so.

#### Distribution.

South Africa (Natal), Lesotho.

#### Habitat and ecology.

In grassland.

### ﻿ var. pubescens

Taxon classificationPlantaeFabalesFabaceae

(E. Phillips) S.A. O’Donnell & G.P. Lewis comb. nov.

D9286B04-F7AB-5248-90CD-1E1D262DB847

urn:lsid:ipni.org:names:77303577-1

#### Type.

SOUTH AFRICA. Natal, Estcourt District, near Little Tugela, 1219 m alt., *J. Medley-Wood 2867* (holotype: NH [NH0002867-0]).

#### Basionym.

ElephantorrhizawoodiiE. Phillipsvar.pubescens E. Phillips, Bothalia 1: 193. 1923.

#### Description.

Stems, petiole, leaf rachis, pinna rachis and inflorescence peduncle and rachis pubescent.

#### Distribution.

South Africa (Natal), Lesotho.

#### Habitat and ecology.

In grassland.

#### Note.

Grobler (2012, p. 151) viewed stem pubescence in *E.woodii* as an unreliable basis for distinguishing these two varieties.

### ﻿ Entada zeylanica

Taxon classificationPlantaeFabalesFabaceae

Kosterm., Misc. Pap. Landbouwhoogeschool 19: 226. 1980.

71F42335-78E4-54F4-BC85-BDEFEA8E803A

#### Type.

SRI LANKA. Southwest Sri Lanka, Sinharaja Forest, *A.J.G.H. Kostermans 26787* (holotype: G; isotypes: K, US [US00170433, US00170434]).

#### Description.

Liana to 50 m long, stem to 50 cm diameter at base; bark greyish-brown, rough, peeling; slash red, fibrous, wood yellow with sparse red sap. **Leaves**: arranged spirally; rachis 8–15 cm long, terminating in a long, strong bifurcating tendril; pinnae 2 pairs per leaf, 5–15 cm long, with 2–4(–5) pairs of leaflets; leaflets 3.5–4.7 × 1.5–2.2 cm, obovate to obliquely oblong, apex obtuse, retuse to emarginate, base acute, lamina glabrous. **Inflorescence**: a spike, 20–22 cm long, axillary, solitary, axis pubescent. **Flowers**: red to dark brown, sessile; calyx reddish-brown, 1–1.5 mm long, glabrous; petals 2.5–3 mm long, green outside, white inside; stamen filaments 3.5 mm long, white. **Fruit**: a torulose, spirally twisted craspedium, 40 × 8 cm, with transverse septa between seeds dividing the fruit into one-seeded segments which, upon ripening, fall from the persistent replum; epicarp woody, endocarp chartaceous. **Seeds**: circular, laterally compressed, concave on both surfaces, 2–3.5 cm diameter, 1.5 cm thick, pleurogram lacking.

#### Distribution.

Sri Lanka.

#### Habitat and ecology.

Wet evergreen rainforest, up to 500 m alt.

## Supplementary Material

XML Treatment for Entada

XML Treatment for Entada abyssinica

XML Treatment for Entada africana

XML Treatment for Entada arenaria

XML Treatment for subsp. arenaria

XML Treatment for subsp. microcarpa

XML Treatment for Entada bacillaris

XML Treatment for var. bacillaris

XML Treatment for var. plurijuga

XML Treatment for Entada borneensis

XML Treatment for Entada burkei

XML Treatment for Entada camerunensis

XML Treatment for Entada chrysostachys

XML Treatment for Entada dolichorrhachis

XML Treatment for Entada elephantina

XML Treatment for Entada gigas

XML Treatment for Entada glandulosa

XML Treatment for Entada goetzei

XML Treatment for subsp. goetzei

XML Treatment for subsp. lata

XML Treatment for Entada hockii

XML Treatment for Entada leptostachya

XML Treatment for Entada louvelii

XML Treatment for Entada mannii

XML Treatment for Entada mossambicensis

XML Treatment for Entada nudiflora

XML Treatment for Entada obliqua

XML Treatment for Entada parvifolia

XML Treatment for Entada pervillei

XML Treatment for Entada phaneroneura

XML Treatment for Entada phaseoloides

XML Treatment for Entada polyphylla

XML Treatment for Entada polystachya

XML Treatment for Entada praetermissa

XML Treatment for Entada rangei

XML Treatment for Entada reticulata

XML Treatment for Entada rheedei

XML Treatment for subsp. rheedei

XML Treatment for subsp. sinohimalensis

XML Treatment for Entada schinziana

XML Treatment for Entada simplicata

XML Treatment for Entada spinescens

XML Treatment for Entada spiralis

XML Treatment for Entada stuhlmannii

XML Treatment for Entada tonkinensis

XML Treatment for Entada tuberosa

XML Treatment for var. tuberosa

XML Treatment for var. pubescens

XML Treatment for Entada wahlbergii

XML Treatment for Entada woodii

XML Treatment for var. woodii

XML Treatment for var. pubescens

XML Treatment for Entada zeylanica
